# Pharmacotherapeutic Considerations on Telomere Biology: The Positive Effect of Pharmacologically Active Substances on Telomere Length

**DOI:** 10.3390/ijms25147694

**Published:** 2024-07-13

**Authors:** Miruna-Maria Apetroaei, Persefoni Fragkiadaki, Bruno Ștefan Velescu, Stella Baliou, Elisavet Renieri, Cristina Elena Dinu-Pirvu, Doina Drăgănescu, Ana Maria Vlăsceanu, Marina Ionela (Ilie) Nedea, Denisa Ioana Udeanu, Anca Oana Docea, Artistidis Tsatsakis, Andreea Letiția Arsene

**Affiliations:** 1 Faculty of Pharmacy, Carol Davila University of Medicine and Pharmacy, 6 Traian Vuia Street, 020956 Bucharest, Romania; miruna-maria.apetroaei@rez.umfcd.ro (M.-M.A.); cristina.dinu@umfcd.ro (C.E.D.-P.); doina.draganescu@umfcd.ro (D.D.); ana.vlasceanu@umfcd.ro (A.M.V.); marina.nedea@umfcd.ro (M.I.N.); denisa.udeanu@umfcd.ro (D.I.U.); andreea.arsene@umfcd.ro (A.L.A.); 2 Laboratory of Toxicology and Forensic Sciences, Medical School, University of Crete, Voutes, 71003 Heraklion, Greece; persefoni.f@gmail.com (P.F.); stellabaliou@gmail.com (S.B.); elisavet_renieri@hotmail.com (E.R.); aristsatsakis@gmail.com (A.T.); 3 Lifeplus S.A., Science & Technological Park of Crete, C Building, Vassilika Vouton, 70013 Heraklion, Greece; 4 Department of Toxicology, University of Medicine and Pharmacy of Craiova, 200349 Craiova, Romania; daoana00@gmail.com

**Keywords:** telomere length, ageing, pharmacotherapy, pharmacotherapy on telomeres, anti-senescence drugs, drugs on telomere length, personalised therapy, novel biomarker

## Abstract

Telomeres are part of chromatin structures containing repeated DNA sequences, which function as protective caps at the ends of chromosomes and prevent DNA degradation and recombination, thus ensuring the integrity of the genome. While telomere length (TL) can be genetically inherited, TL shortening has been associated with ageing and multiple xenobiotics and bioactive substances. TL has been characterised as a reliable biomarker for the predisposition to developing chronic pathologies and their progression. This narrative review aims to provide arguments in favour of including TL measurements in a complex prognostic and diagnostic panel of chronic pathologies and the importance of assessing the effect of different pharmacologically active molecules on the biology of telomeres. Medicines used in the management of cardiovascular diseases, diabetes, schizophrenia, hormone replacement therapy at menopause, danazol, melatonin, and probiotics have been studied for their positive protective effects against TL shortening. All these classes of drugs are analysed in the present review, with a particular focus on the molecular mechanisms involved.

## 1. Introduction

Telomeres are specialised nucleoprotein structures located at the ends of linear chromosomes. Their main function is to inhibit the activation of the response to DNA damage [1]. Telomeres are chromatin structures containing repeated DNA sequences that function as protective caps at the ends of chromosomes and prevent DNA degradation and recombination, thus ensuring the integrity of the genome [2].

**Figure 1:**
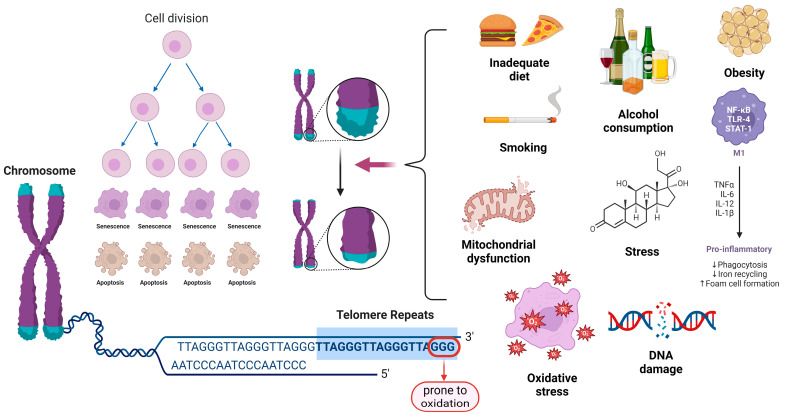
Physiological, lifestyle, and environmental causes contributing to TL shortening. Legend: IL-6—interleukin 6; IL-12—interleukin 12; IL-1β—interleukin 1β; TNFα—tumour necrosis factor alpha; DNA—deoxyribonucleic acid; ↓—decrease; ↑—increase.

Telomerase, which functions mainly as a reverse transcriptase capable of maintaining TL, adds short repeat sequences to the ends of chromosomes, compensating for the inherent loss of DNA in genome replication. The telomerase RNA component and the telomerase reverse transcriptase (TERT) protein are the main components of telomerase. Additionally, telomerase activity is regulated by multiple proteins that attach to one of the active components [3]. As they shorten, telomeres no longer have the ability to attach enough telomere-capping proteins. This exposes the final portions of the DNA and triggers the DNA denaturing response pathways, which, by inducing the cell cycle inhibitors p21 and p16, block proliferation [4]. Figure 1 illustrates an overview of the factors that contribute to TL shortening.

While TL can be genetically inherited, TL maintenance has been associated with multiple xenobiotics and bioactive substances. This can be attributed both to direct protection mechanisms against factors that contribute to oxidative stress, inflammation, and mitochondrial dysfunction and to an effect on telomerase activity [5]. Telomere shortening is a characteristic feature of the physiological ageing process and is closely related to other key aspects associated with ageing, including cellular senescence, stem cell depletion, genome instability, disruption of epigenetic control, mitochondrial instability, and inflammation [6,7].

TL has been characterised as a reliable biomarker for the predisposition to chronic pathologies and their progression. These include cardiovascular diseases [8], metabolic diseases [9], osteoporosis and osteoarthritis [10], neurodegenerative diseases [11], psychiatric diseases [12], male or female infertility [13], and cancer [14]. Patients with non-communicable chronic diseases require effective pharmacotherapy to obtain optimal clinical results and improve their quality of life. Most of them present multiple comorbidities associated with complex therapy regimens [15].

The evaluation of TL as a biomarker included in a complex prognostic and diagnostic panel of chronic pathologies should be implemented, considering the patients’ medication. Pharmacologically active substances can have a protective effect against the aberrant shortening of TL associated with chronic diseases, while other drugs can have an impact on accelerating telomere erosion. This narrative review aims to analyse the molecular mechanisms by which certain classes of drugs can inhibit TL shortening. Understanding these aspects offers promising perspectives for improving the results of the pharmacotherapeutic regimen and increasing health outcomes.

## 2. Molecular Mechanisms of Ageing

Ageing is an inherent biological process characterised by the gradual decline of physical condition, leading to the impairment of several physiological functions [16]. This process is both ubiquitous and unavoidable. Specific alterations are harmless, such as the greying of hair, while others lead to a decrease in the functioning of the senses and the ability to perform daily activities, as well as a greater vulnerability to sickness, weakness, and disability [17].

Ageing is a result of the gradual reduction in the TL, programmed cell death, or the development of cancerous cells in non-reproductive cells, which impacts the overall well-being and duration of life of an individual. There is a correlation between shorter telomeres and higher rates of illnesses and worse survival rates [18]. Cellular senescence, which has significant impacts on the regulation of normal tissue balance and the development of diseases, is another primary contributing component to the ageing process and the onset of age-related ailments [19]. Senescence triggers initiate targeted modifications in intracellular processes in order to establish a durable cessation of the cell cycle. The initiation of this process occurs at the *INK4A-ARF* locus, which contains genes that suppress tumours and is located in the chromosomal region 9p21. The expression of the gene at the specific location is often suppressed by Polycomb Repressive Complexes 1 and 2 (PRC1 and 2). The breakdown of PRC1/2 leads to the activation of genes and the transcription of two distinct proteins, including p16^INK4A^ and p14^ARF^. These abnormalities are linked to the development of cancer and activate oncogene-induced senescence as a preventive measure [20].

Recent findings indicate that ageing is strongly linked to altered communication between cells, damage to DNA, exhaustion of stem cells, depletion of nicotinamide adenine dinucleotide levels, dysfunction of mitochondria, imbalance of protein levels, impaired macro-autophagy, inflammation, disrupted nutrient sensing, and an imbalance in the gut microbiota [21]. Moreover, the process of ageing is propelled at the cellular level by stochastic molecular harm that gradually builds up over time. While cells do have processes to repair or eliminate damage, their effectiveness is not perfect and decreases as they age [22]. Epigenetic changes, including alterations to histone chromatin remodelling and DNA methylation, gradually occur in the cells of ageing individuals. These changes are linked to ageing characteristics and the onset of age-related disorders [23]. Given that genomic mutations are irreversible, whereas epigenetic modifications can be reversed, targeting the reversal of epigenetic modifications is a viable strategy for treating cells with the goal of postponing ageing [24].

In contrast to the process of telomere shortening, which initiates cellular ageing, telomerase reverses this process by restoring missing DNA sequences throughout cell division, thereby adding time to the molecular clock and increasing the cell’s lifespan [25]. Nevertheless, the quantity of telomerase decreases following each cell division [26]. Therefore, modern medicine has been focusing on finding potent telomerase activators in order to slow down the ageing process [27].

Besides telomerase activation, there exists an alternate method for lengthening telomeres known as ALT (alternative lengthening of telomeres). However, this process is only found in aberrant settings, such as in cancer cells, immortalised cell lines, and mouse cell deletion for the telomerase gene. Normal human lymphocytes are unable to utilise ALT to preserve their telomeres [26,28]. Basically, ALT tumours maintain their ability to divide indefinitely by lengthening their telomeres throughout the G2 and M stages of the cell cycle using a specific break-induced replication pathway. This can clarify the reasons behind therapeutic failures and resistance to anti-cancer therapy that is based on telomerase suppression [29].

Degenerative pathologies facilitate the ageing process by inducing a progressive accumulation of mutations, preventing cell division, and making cells more vulnerable to apoptosis. Basically, human cells have the ability to divide a finite number of times until they reach a state called senescence, when division is no longer possible [26]. Cellular senescence is triggered by internal and external stressors such as activation of oncogenes, telomere dysfunction, and long-term DNA damage. The extrinsic mechanisms of senescent cells, generally characterised by the amplification of the secretory phenotype associated with senescence, intensify the intrinsic proliferative arrest inside a cell and contribute to the development of pathologies related to ageing and defective tissue regeneration [30,31]. Elimination of senescent cells may reduce tissue dysfunction associated with ageing, while senescence, in some cases, may serve as a potent anti-tumour mechanism by inhibiting the growth of potentially malignant cells [32].

## 3. Pharmacologically Active Substances Acting on the Cardiovascular System

Cardiovascular diseases (CD) continue to represent a primary factor in premature death while increasing the costs of health systems. Various factors lead to the burden of CD, including environmental, lifestyle, cardiometabolic, and social aspects [33,34]. Pharmacotherapeutic regimens for CD management target the main risk factors, namely obesity, diabetes, hypertension, dyslipidaemia, and smoking. Even so, a large number of patients acquire CD without presenting these risk factors. For this reason, the exact understanding of the pathological mechanisms involved in the development of CD has been insufficient until now [35]. Various empirical studies have demonstrated an important correlation between reduced TL and increased cardiovascular risk [36,37,38].

Telomere shortening and dysfunction are etiological factors in the development and aggravation of CD associated with ageing [38]. Excessively shortened telomeres trigger cell senescence, followed by apoptosis, and have been identified as a biomarker in the progression of arteriosclerosis and arterial plaque instability [39]. In practice, TL serves as a credible indicator of the combined impact of oxidative stress and inflammation accumulated during life. An aberrantly shortened TL has been attributed to increased susceptibility to CD, a greater chance of developing cardiovascular risk factors, and the possibility of sudden death from a cardiovascular cause [39,40]. The close causal relationship between a reduction in TL in different types of cells and the presence of atherosclerosis, ischemic cardiovascular disease, myocardial infarction, and sudden death from cardiovascular causes suggests that average TL and telomerase activity are important biomarkers in cellular ageing [41].

Insufficient blood pressure control, determined in the ambulatory over 24 h, was attributed to a low serum concentration of telomerase reverse transcriptase, an unfavourable metabolic profile of adipose tissue, and an aberrant endothelium function [42]. Moreover, by analysing the data obtained in the Framingham study, it was concluded that the renin-angiotensin system (RAS), characterised by a high concentration of the renin-angiotensin ratio in serum, has an increased prevalence of shortening TL in patients with arterial hypertension [43]. Therefore, it was concluded that therapeutic agents that intervene in the RAS present therapeutic opportunities for efficiently controlling blood pressure, improving patient survival rates, and protecting telomeres from the factors involved in their shortening. A study that included 156 patients with type II hypertension of whom 96 had type 2 diabetes as a comorbidity, hypothesised that patients with double comorbidity have a much shorter TL. Achieving optimal blood pressure through pharmacotherapy has proven more effective in preserving TL than efficient blood sugar control [44].

The physiological ageing process is associated with multiple changes in the body’s functioning, structure, and physiological mechanisms, all of which are very similar to the changes induced by hypertension [19]. The components of the circulatory system are subject to multiple changes, such as vascular remodelling, inflammation, increased stiffness, endothelial dysfunction, and calcification. The molecular mechanisms and cellular processes that cause vascular alterations both in the case of hypertension and in the case of the physiological ageing process include oxidative stress, an abnormal transmission of signals, and the activation of transcription factors that promote inflammation and fibrosis [45]. Thus, hypertension and the cardiovascular changes caused by it represent a critical therapeutic target for CD management and for preserving TL. At the same time, the pharmacologically active drugs used are the main pillars for obtaining optimal results.

### 3.1. Statins

Statins are first-line drugs that reduce the risk of cardiovascular events and are considered the gold standard for the treatment of dyslipidaemia. Hydroxy-methyl-glutaryl coenzyme A (HMG-CoA) reductase catalyses the transformation of HMG-CoA into mevalonic acid, a critical step in the production of endogenous cholesterol. Thus, statins reduce cholesterol synthesis in the liver by competitively inhibiting the HMG-CoA enzyme [46,47,48]. In addition to their increased potency as lipid-lowering drugs, statins possess a series of pleiotropic actions independent of their main action. The most studied pleiotropic effects are antioxidant activity, rebalancing endothelial function, stabilising the atherosclerosis plaque, anti-inflammatory effects, protective effects in the progression of neurological disorders, and antithrombotic action [49,50].

Statin treatment has been reported to impact the length of telomere G-tails. Thus, statins are hypothesised to prevent the aberrant shortening of telomeres at a molecular level by interacting with the telomere/telomerase system and combating oxidative stress [51]. Statins regulate cellular pathways of oxidation that govern the activity of nicotinamide adenine dinucleotide phosphate (NADPH) oxidase, endothelial nitric oxide synthase (eNOS), and myeloperoxidase [52]. This modulation promotes an antioxidant effect that restores the endogenous redox balance [53]. At the same time, statins influence the signalling of nuclear factor erythroid 2 related factor 2 (Nrf2) and heme oxygenase-1 (HO-1), leading to cellular defence against reactive oxygen species [53,54]. These molecules substantially enhance the ability of Nrf2 to bind to DNA and stimulate HO-1 and glutathione peroxidase activation. Thus, through the phosphoinositide 3-kinase/protein kinase B (PI3K/Akt) cellular pathway, statins activate Nrf2, an effect that culminates in inhibiting the formation of reactive oxygen species [55].

**Figure 2:**
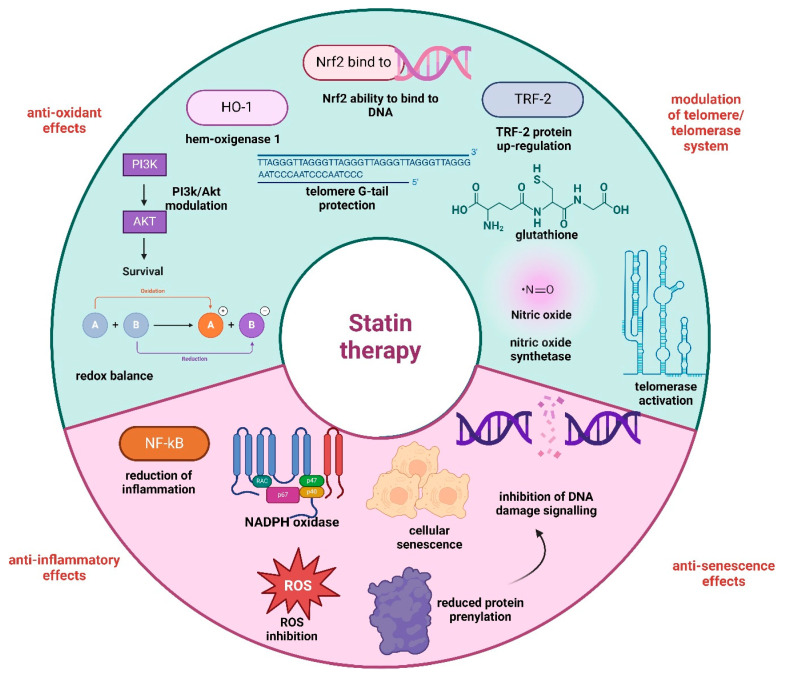
Molecular mechanisms of statin therapy on telomere maintenance. Legend: PI3k/Akt–phosphoinositide 3–kinase/protein kinase B; Nrf2–nuclear factor erythroid 2 related factor 2; TRF-2–telomeric repeat-binding factor 2; NF-kB–nuclear factor kappa-light-chain-enhancer of activated B; NADPH oxidase–nicotinamide adenine dinucleotide phosphate oxidase; ROS–reactive oxygen species; DNA–deoxyribonucleic acid.

Various cells, such as vascular smooth muscle cells, endothelial cells, endothelial progenitor cells, and chondrocytes, can undergo cellular senescence, while statins effectively counteract this process [56]. Reducing oxidative stress, promoting increased glutathione synthesis, inhibiting protein prenylation that induces DNA damage, inhibiting subsequent signalling from impaired DNA, and accelerating DNA repair are all potential ways statins protect against DNA damage [57]. Moreover, the telomere-capping protein TRF2, which helps stabilise the telomeric structure, has its expression upregulated by statins. Increased TRF2 expression in the endothelium and other relevant cells may be responsible, at least in part, for the reduction in clinical events in patients with shorter telomeres using statins. This is mainly due to the fact that telomere dysfunction might be caused by a loss of TRF2 [56,58]. Figure 2 illustrates some of the molecular mechanisms of statin therapy in telomere biology.

A cross-sectional study that included 3496 participants found that statin therapy directly correlates with increased telomerase activity [58]. Additionally, according to the latest clinical studies, statin therapy can decrease the frequency of clinical events [59,60]. Still, this effect is limited to the case where TL drastically endangers the patients’ lives. This provides solid arguments in favour of using TL as a marker in diagnosing cardiovascular diseases and may serve as a valuable tool for physicians to divide critical patient categories and appropriate regimens based on TL [61]. In a study conducted by Bennaceur et al., a 6-fold increase in telomerase activity was seen in human and mouse peripheral blood mononuclear cells (PBMCs) and CD4 T cells after being treated with atorvastatin at concentrations ranging from 0.1 to 0.3 μM, which resulted in modest proliferation of T lymphocytes. Telomerase activity was disabled, and proliferation was entirely reduced by high doses of atorvastatin (2–5 μM) or LDL cholesterol. The proliferative effects of atorvastatin were abolished in the absence of telomerase reverse transcriptase (TERT). During the initial five months, the percentage of telomerase-positive lymphocytes in transgenic GFP-mTert reporter mice dropped from 30% to 15%. As a result, the authors concluded that, throughout the course of typical development and maturation, lymphocyte telomerase activity declines in vivo alongside immune cell turnover [62]. After controlling for chronic inflammation and oxidative stress markers, atorvastatin treatment remained the sole independent predictor of telomerase activity changes in a multiple-regression analysis. Treatment with atorvastatin was linked to normal-range increases in interleukin-6 (IL-6) and a trend towards decreased blood urea. Based on these preliminary findings of a study conducted by Strazhesko et al., atorvastatin may have a role as a geroprotector and telomerase activator [63].

### 3.2. Calcium Channel Blockers (CCB)

Drugs from the class of calcium channel blockers (CCB) are divided into two categories: dihydropyridines and non-dihydropyridines. These have multiple pharmacotherapeutic indications, including arterial hypertension, angina pectoris, hypertrophic cardiomyopathy, pulmonary hypertension, and supraventricular arrhythmia [64]. By inhibiting calcium entry into cells, CCB helps lower blood pressure and can be used with other pharmacotherapeutic regimens. CCBs of the non-dihydropyridine type show more pronounced chronotropic and inotropic effects than the dihydropyridine ones, especially relevant for patients with supraventricular arrhythmias [65]. For these reasons, CCBs are recommended as the first-line option in the treatment of hypertension [66,67].

Regarding the molecular mechanisms on telomeres, CCBs have been identified as having a protective effect on TL through a mechanism dependent on eNOS, which gives them an effect against cellular senescence [68]. CCB possesses antioxidant activity at the level of cellular structures and prevents the inactivation of telomerase, increasing the activity of eNOS during the vascular endothelial senescence process [69]. A study by Tand et al. showed that patients using CCB showed significant reductions in DNA-methylation and functional biological ages, in contrast to those who did not take these medications. The conclusion implies that CCBs may have the capacity to decrease biological ageing, as indicated by the biomarkers analysed in the study [70]. Moreover, some positive correlations were drawn between TL and the decrease in systolic blood pressure and pulse, especially in the case of patients under treatment with CCB and ARB [71].

### 3.3. Agents Acting on the Renin-Angiotensin System (RAS)

RAS is involved in maintaining homeostasis and controlling vasoconstriction. However, this system is responsible for causing fibrosis, inducing inflammation, and oxidative stress. The main product of RAS is angiotensin II, which was discovered to be involved in developing chronic pathologies associated with ageing [72,73]. Angiotensin II binds to the angiotensin type 1 receptor (AT1R), induces physiopathological changes accompanied by marked oxidative stress, and is linked to mitochondrial dysfunction and telomere erosion [74].

**Figure 3:**
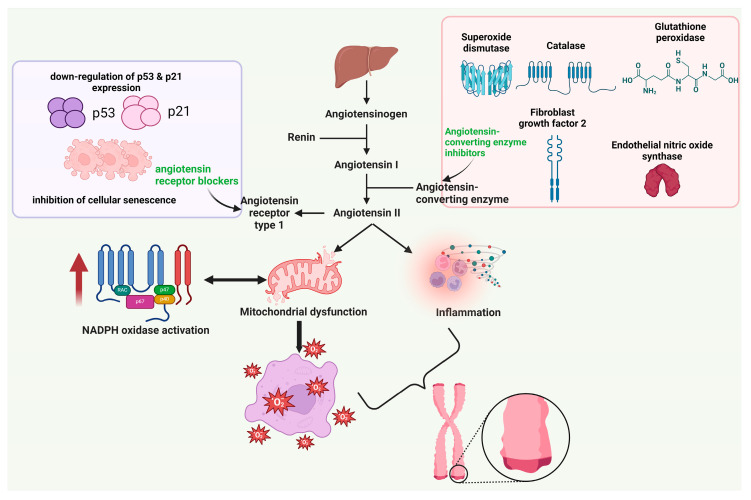
RAS on TL. Legend: ACE—angiotensin-converting enzyme; AT1R—angiotensin II receptor type 1; NADPH oxidase—nicotinamide adenine dinucleotide phosphate oxidase; ARB—angiotensin receptor blockers; ACEi—angiotensin-converting enzyme inhibitors; ↓—decrease, ↑—increase.

In this direction, the development of pharmacologically active agents capable of influencing the RAS represented a new therapeutic opportunity. AT1R antagonists (ARB) such as losartan, candesartan, telmisartan, etc., and angiotensin-converting enzyme inhibitors (ACEi) such as captopril, lisinopril, enalapril, etc., can restore the functioning of endothelial progenitor cells by facilitating communication between telomerase enzymes [75]. Figure 3 depicts the RAS’s involvement in TL shortening and the mechanisms by which ARB and ACEi counteract these effects.

The protective action of ARB on cognitive decline associated with cerebral vascular aneurysms and ageing has been demonstrated in clinical studies [76,77]. Several authors have concluded that blocking the RAS with antagonistic pharmacological agents is an optimal technique to slow the physiological ageing process [74,78]. In the Framingham Heart Study, Vasan et al. highlighted that adults with an increased renin-angiotensin ratio, characteristic of patients with hypertension, have a shorter TL [43]. Another study determined the TL of pregnant women through natural pregnancy, in vitro fertilisation, and intracytoplasmic sperm injection. The conclusion emphasised an inversely proportional correlation between serum renin concentration in the first trimester and TL, as women who became pregnant through in vitro fertilisation or intracytoplasmic sperm injection had shorter TL than those who experienced a naturally occurring pregnancy [79]. Both murine models of arterial hypertension and in vitro studies have emphasised the negative effect of RAS on TL and the fact that pharmacological agents that intervene in this system manage to offer additional protection against telomere erosion [80]. In an in vivo study, the group treated with a combination of angiotensin II and losartan showed increased TL, decreased staining of β-galactosidase, and reduced expression of p53 and p21 compared to the group treated with angiotensin II alone. This study validates the hypothesis that angiotensin II triggers the reduction in TL, the production of p53 and p21, the halting of the cell cycle, and the consequent cellular senescence. Furthermore, losartan effectively decreased the rate at which telomeres shorten and prevented cellular senescence [81]. Fyhrquist et al. analysed two consecutive DNA samples from 132 patients with type 1 diabetes. This study concluded that short TL is a predictive biomarker for the development and progression of type 1 diabetes. In addition, the authors outlined that patients who followed an ARB or ACEi therapy had longer TL than patients using other classes of anti-hypertensive drugs such as diuretics, beta-adrenergics, or calcium channel blockers [82].

It is noteworthy that angiotensin-converting enzyme inhibitors have an important advantage over angiotensin receptor blockers. ACEi offer additional vascular protection due to their ability to increase the tissue concentration of bradykinin by inhibiting its degradation, which is particularly important for coronary artery disease [83]. At the level of endothelial cells, bradykinin activates the antioxidant enzymes catalase, superoxide dismutase, and glutathione peroxidase [84]. At the same time, by increasing the level of eNOS, fibroblast growth factor 2, and TERT messenger RNA and possessing antioxidant properties, ACEi help restore the activity and increase the survival of endothelial cells caused by a vascular injury [58,85]. De Vries et al. and Akinnibosun et al. emphasised that early initiation of ACEi therapy positively impacts systolic blood pressure and TL. Moreover, ACEi are hypothesised to exert epigenetic modifications closely related to the protection against arterial hypertension [86,87].

## 4. Pharmacologically Active Substances Used in Diabetes

Diabetes remains one of the most widespread non-communicable diseases worldwide, with a high prevalence, reaching a number of 2 million deaths in 2019 [88]. Diabetes is a pathology that affects multiple organs, with the most common chronic complications being diabetic foot, diabetic retinopathy, osteoporosis, arthropathies, decreased immunity manifested by an increased incidence of infections, and diabetic neuropathy. Additionally, acute complications such as diabetic ketoacidosis are life-threatening and require immediate hospitalisation and support of vital function [89,90,91].

TL is of particular interest concerning diabetes, which is a condition characterised by accelerated cellular ageing caused by a complex interplay of genetic and environmental factors [7,9]. Patients with diabetes present a shorter TL, but the exact mechanisms have not yet been clearly established. However, this may be influenced either by hyperglycaemia per se, by the associated oxidative stress, or by the accumulation of metabolic toxins [92]. Moreover, insulin resistance, obesity, and hyperinsulinemia lead to increased oxidative stress and, implicitly, to the shortening of TL [93,94]. In a meta-analysis conducted on 17 cohorts, including over 5500 patients diagnosed with type 1 and type 2 diabetes, the authors identified that diabetic patients had a much shorter TL compared to healthy controls. Moreover, it is noteworthy that TL shortening was pronounced in the case of type 2 diabetes compared to type 1 diabetes and in the case of patients younger than 60 years old with type 2 diabetes [95]. Additionally, a study conducted by Baltzis et al. on 90 diabetic patients with diabetic ulcers concluded that among this population, besides having a higher neuropathy impairment score and markedly larger waist size, the activity of telomerase was aberrantly reduced [96].

It is well established that elevated glucose levels increase the generation of reactive oxygen species and induce dangerous reactions that affect different cellular signalling pathways [97,98]. All these cause single-chain breaks in DNA and erosion of telomeres, which in turn cause senescence of β-pancreatic cells and a decrease in the mass of these cells, insulin production, and glucose tolerance. Consequently, the generated oxidative stress causes impaired activity of the telomere-telomerase system, thus creating a vicious circle [94,99]. At the same time, hyperglycaemia increases the generation of reactive oxygen species at the level of the electron transport chain in the mitochondria through the formation of complex end products of glycation and the increase in glucose auto-oxidation. The guanine pairs present in telomeres are even more prone to oxidation, especially during mitosis, where they exist in solitary cells, making them vulnerable to oxidation and destruction [94,100,101]. In a study by Monickaraj et al., a causal relationship was drawn between mitochondrial dysfunction and diabetes. In the case of patients with type 2 diabetes, a marked increase in lipid peroxidation, a much-shortened TL, and a decrease in adiponectin levels and mitochondrial DNA content were observed [102]. Another study hypothesised that type 2 diabetes could cause epigenetic changes at the level of telomeric structures through DNA methylation at the level of long interspersed element-1 (LINE-1) [103].

However, pharmacotherapeutic regimens used in diabetes management offer promising perspectives to mitigate the impact of telomere shortening [104]. Hypoglycaemic drugs such as metformin, sulfonylurea, and dipeptidyl peptidase-4 inhibitors have been proven to mitigate the changes triggered by oxidative stress on telomeres. However, insulin in the form of injectable preparations administered in type 1 diabetes or in type 2 diabetes that is refractory to antihyperglycemic medication has been shown to accelerate telomere attrition [105,106].

### 4.1. Biguanides

**Figure 4:**
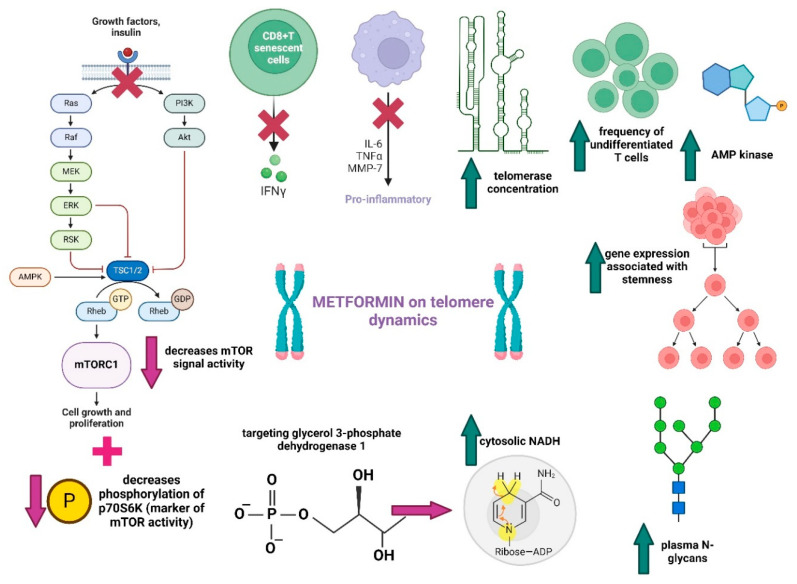
Metformin as a prospective anti-ageing drug. Legend: mTOR—mammalian target of rapamycin; Raf—rapidly accelerated fibrosarcoma; MEK—mitogen-activated and extracellular-signal-regulated kinase; ERK—extracellular-signal regulated kinases; RSK—ribosomal S6 kinase; AMPK—adenosine monophosphate-activated protein kinase; TSC1/2—tuberous sclerosis proteins 1 and 2; Rheb—Ras homolog enriched in brain; mTORC1—mammalian target of rapamycin complex 1; GTP—guanosine triphosphate; P—phosphorylation; p70S6K—p70 S6 kinase; PI3k/Akt—phosphoinositide 3-kinase/protein kinase B; IFNγ—interferon-gamma; IL-6—interleukin 6; TNFα—tumour necrosis factor alpha; MMP-7—matrix metallopeptidase 7; NADH—nicotinamide adenine dinucleotide; ↓—decrease; ↑—increase.

Metformin is a pharmacologically active agent from the biguanide class used as the first-line treatment option for type 2 diabetes, having efficacy both as monotherapy and as a combined regimen with other antihyperglycemic agents. The clinical applications of metformin have evolved from its first indication as a drug for influenza to the gold standard in type 2 diabetes and continue to develop until now [107]. Recent studies have demonstrated the prospective potential of this drug for multiple clinical applications, including as an anti-ageing agent [108,109,110]. Figure 4 provides an overview of the pathways employed by metformin to counteract ageing.

Metformin promotes insulin sensitivity and restores normal IGF-1 levels by stimulating AMPK and inhibiting the signal activity on the mTOR pathway [111]. These mechanisms have considerable clinical utility since mTOR signalling leads to accelerated ageing, and changes in this pathway are associated with cancer, inflammation, and neurological diseases [112,113]. Metformin, in low doses, targets the lysosomal AMPK pathway through presenilin enhancer 2 (PEN2) and hepatic gluconeogenesis, in addition to other AMPK-independent pathways, such as inhibition of mitochondrial glycerophosphate dehydrogenase [114,115].

It is hypothesised that the association of metformin with its target glycerol 3-phosphate dehydrogenase (GPD1) can partly explain its anti-ageing effect. Metformin inhibits mitochondrial GPD1, blocks lactate-glucose incorporation, and enhances cytosolic NADH [116]. It has been shown that there is an overexpression of GPD1 in long-living organisms, while a decrease in GPD1 levels inhibits replicative life cycles. Additionally, overexpression of GPD1 by metformin underlines the drug’s anticancer activity in vitro [117].

In the same direction, in a recent study by Yang et al., metformin reduced the number of senescent CD8+ T cells. Metformin decreased the release of IFNγ from senescent CD8+T cells (through an effect on the senescence-associated secretory phenotype), decreased the production of proinflammatory cytokines IL-6, increased the synthesis of TNFα in senescent cells, increased the concentration of telomerase, increased the frequency of undifferentiated T cells, enhanced the expression of genes associated with stemness and those associated with telomerase activity, and decreased the expression of genes associated with DNA damage [118].

In a randomised, double-blind study that included 38 diabetic patients, metformin increased silent information regulator sirtuin 1 (SIRT1) gene expression, SIRT1 chromatin promoter accessibility, SIRT1 protein synthesis, and decreased p70S6K phosphorylation while having a positive effect on plasma N-glycans [119]. SIRT1 reduces the production of inflammatory cytokines by directly inhibiting the transcription of target genes through the deacetylation of histones in the promoter region of those genes [120]. Thus, metformin represents a prospective anti-ageing drug that interacts with multiple pathways involved in longevity and maintaining the integrity of telomeres.

### 4.2. Dipeptidyl Peptidase-4 Inhibitors (DPP-4i)

DPP-4i elicits a significant decrease in serum glucose levels, which is relevant in the clinical context of type 2 diabetes, especially as it presents a low risk of hypoglycaemia and does not cause weight gain. At the same time, DPP-4i has beneficial effects independent of the hypoglycaemic action, such as lowering systolic blood pressure, reducing total cholesterol and triglycerides, and increasing the activity of β-pancreatic cells [121,122].

Therefore, the pharmacodynamic effects of this class represent a pertinent approach for repurposing them as agents that could contribute to protecting telomeres from erosion. Dudinskaya et al. conducted a clinical study to determine whether vildagliptin-metformin combined therapy could provide superior benefits in inhibiting telomere attrition compared to metformin monotherapy. Both therapies proved effective for optimal glycemic control. In contrast, patients who received the combined therapy experienced a statistically significant increase in telomerase activity, thus concluding that the metformin-vildagliptin combination could have a new pleiotropic effect by modifying telomerase activity [123].

## 5. Pharmacologically Active Substances Acting on the Central Nervous System

### 5.1. Antipsychotics

Schizophrenia (SCZ) is a complex neurological disorder, presenting multiple dysfunctional brain regions and few efficient management methods [124]. First-generation antipsychotic medications are defined by their ability to block dopamine D2 receptors. Second-generation antipsychotic medications, in contrast, act as serotonin and dopaminergic receptor agonists. Although this class varies greatly in terms of adverse reactions, in terms of therapeutic benefit, there are insignificant variations [125].

In SH-SY5Y cells, aripiprazole has shown neuroprotective benefits by raising brain-derived neurotrophic factor transcripts and proteins, phosphorylation, and GSK-3β, and lowering synaptosome presynaptic-like glutamate release [126]. Brain-derived neurotrophic factor presents a protective effect against oxidative damage in neurons, as it increases the expression levels of manganese superoxide dismutase, which in turn enhances the antioxidant capacity of the cell [127]. Aripiprazole stands out among antipsychotics due to its specific ability to partially agonist both dopamine D2 and serotonin 5-HT1A receptors [126]. When compared to traditional antipsychotics, atypical antipsychotics have a reduced incidence of certain causes of death and extrapyramidal symptoms [128]. Stroke and metabolic syndrome, both of which can cause metabolic changes, are more common in patients taking atypical antipsychotics compared to those taking conventional antipsychotics [129]. Second-generation antipsychotics, especially clozapine, have been reported to have a strong antioxidant effect, counteracting oxidative stress caused by aberrant activation of microglia. This marked reduction in free radicals provides a neuroprotective effect against oxidative stress induced by activated microglia [130,131].

Various environmental and genetic factors contribute to the development and progression of SCZ, which has led to the hypothesis that this pathology is a condition characterised by accelerated ageing, associated with physiopathological changes also present in the case of physiological ageing. Due to the marked oxidative stress in the case of SCZ, telomere erosion can be accelerated, which has been demonstrated in many instances of SCZ patients [124,132]. Table 1 summarises some of the most recent studies regarding the correlation between SCZ and TL.

Studies show contradictory conclusions regarding a positive correlation between TL and the presence of schizophrenia. Interestingly, in most studies, it was concluded that patients who received treatment with antipsychotic drugs had a longer TL than those naïve to antipsychotic therapy [126,135,136,140]. At the same time, two studies have concluded that antipsychotic drugs have no effect on TL [138,139]. An interesting finding is represented by the protection offered by antipsychotic medication in vitro following a chemical exposure to oxidative stress [126]. This underlines the antioxidant effect of antipsychotic medication discussed in the literature.

### 5.2. Melatonin

The pineal gland produces a single hormone, secreted in the absence of daylight. Melatonin is synthesised from serotonin, derived from the amino acid tryptophan, through a series of enzymatic processes in which, in the last two stages, the enzymes arylalkylamine N acetyltransferase and hydroxyl-indole-O-methyltransferase are involved. From the postganglionic fibres, the gland receives stimuli that increase the synthesis of cyclic AMP and trigger the release of noradrenaline, which activates the enzyme arylalkylamine N acetyltransferase. After the endogenous synthesis, this neurohormone is released into the blood and reaches all tissues [142,143,144].

The inversely proportional relationship between endogenous melatonin concentration and sleep quality is accentuated with physiological ageing, and for this reason, it has been hypothesised that melatonin deficiency contributes to the development and progression of sleep disorders [145,146]. Melatonin reduces the interval of falling asleep, increases sleep duration, and significantly decreases the number of nocturnal awakenings. Therefore, melatonin in the form of food supplements is often administered for incipient sleep disorders [147,148,149].

Melatonin exerts its actions through receptors coupled to protein G located at the level of cell membranes. MT1, MT2, and MT3 are found in almost all human body tissues. The MT1 receptor is coded by chromosome 4 and has 351 amino acids; the MT2 receptor is coded by chromosome 11 and continues with 363 amino acids; and MT3 shows a very high structural similarity with human quinone reductase 2, the enzyme involved in the detoxification process. Thus, MT3 inhibits leukocyte adhesion mediated by leukotriene B4 and reduces intraocular pressure [150,151].

Melatonin has anti-inflammatory and antioxidant actions due to its metabolites, N1-acetyl-N2-formyl-5-methoxykynuramine and N1-acetyl-5-methoxykynuramine. Consequently, melatonin helps maintain cellular integrity by neutralising free radicals that lead to lipid peroxidation and contributing to the transcription of genes that encode glutathione peroxidase, as well as other enzymes with antioxidant actions [152]. Moreover, it has anti-ageing action at the level of blood vessels, attributed to the direct detoxification of free radicals and the maintenance of oxygen metabolism in the mitochondria [153]. In the same direction, melatonin stimulates the production of antibodies and modulates immune functions, including the function of defence against tumours, acting as an immunomodulatory agent [152].

A suboptimal sleep quality, characterised by a low melatonin concentration, was associated with an aberrant secretion of cortisol and cytokines [154]. This evidence is the basis of the hypothesis that a marked decrease in melatonin synthesis could cause increased oxidative stress that culminates with telomere erosion. For this reason, exogenous supplementation with melatonin could have a protective effect on TL and mitigate oxidative stress [155,156].

In a murine model of atherosclerosis in which mature animals presented vascular endothelial lesions, an increase in the concentration of proinflammatory cytokines, reactive oxygen species, superoxide dismutase, and malondialdehyde was observed. The administration of exogenous melatonin led to a decrease in these markers. Also, melatonin counteracted the effects caused by H_2_O_2_ and reduced vascular endothelial damage by modulating telomerase activity [157]. In an in vitro study, Liu et al. demonstrated that melatonin contributes to improving the DNA’s self-repair capacity by influencing several genes that are involved in the pathways responsible for DNA denaturation [158].

## 6. Pharmacologically Active Substances Acting as Modulators of the Genital System

### 6.1. Hormone Replacement Therapy

Oestrogen receptors belong to the nuclear hormone receptor superfamily and function as ligand-dependent transcription factors. Oestrogens exert their actions by interacting with the ligand-dependent receptors ER-α and ER-β, which are essential in tissue growth and differentiation [159,160]. It is interesting to note that although the withdrawal of oestrogens leads to atrophic changes, continuous exposure in large quantities leads to tumour development at the breast and ovarian levels [161,162].

Many lines of evidence support the fact that oestrogen has direct and indirect control over telomerase in tissues that are sensitive to these hormones, thus maintaining the integrity of telomeres [161,163]. Lin et al. found that extended exposure to endogenous oestrogen was linked to increased TL and decreased telomerase activity. Moreover, the duration of reproductive years was adversely related to the combination of a short TL and high telomerase activity. Therefore, the authors concluded that endogenous oestrogens could potentially slow down the process of cellular ageing [164]. Schuermans et al. demonstrated that an earlier onset of menopause is linked to a lower TL, particularly in women who have natural menopause. The accelerated shortening of TL during premature menopause may be attributed to an increased CD risk. Researchers Park et al. found that estrogen treatment of mice reverses the ageing-associated reduction in beige adipogenesis. The researchers discovered that nicotinamide phosphoribosyl transferase (NAMPT) is necessary for the production of E2-induced beige adipocytes, which in turn prevents the development of age-associated stress within the endoplasmic reticulum. Also, by increasing the number of perivascular adipocyte progenitor cells, NAMPT signalling can be achieved through genetic or pharmacological means, allowing for the restoration of beige adipocyte production [165]. On the other hand, progesterone and growth factors such as TGF-β inhibit telomerase activity and hTERT gene production [161].

In most countries worldwide, the life expectancy of women is higher than that of men, which can be explained by the differences in TL. Although there is no significant difference between TL at birth in men and women, as the body matures, men show a much more considerable TL shortening than women. A possible mechanism to explain this is represented by the presence of oestrogen hormones in females, which leads to lower oxidative stress in this population and a higher activity of telomerase [166,167].

Menopausal replacement therapy is frequently prescribed to alleviate symptoms associated with menopause. Treatment options can be estrogen-progesterone combined therapy or estrogen-only therapy. Oestrogen can be administered in various pharmaceutical forms, such as oral, transdermal, percutaneous, intramuscular, intranasal, subcutaneous, and vaginal, while the dose is customised depending on the patient [168,169]. Table 2 represents an overview of empirical data assessing the impact of antipsychotics on TL.

### 6.2. Antigonadotropins (Danazol)

Endometriosis is a condition that affects over 190 million women of reproductive age and is characterised by persistent and debilitating pain, pelvic distress, nausea, and exhaustion and can lead to depression, anxiety, and infertility. From a physiopathological point of view, endometriosis is characterised by the proliferation of endometrial cells on extraneous tissues such as the ovaries or fallopian tubes [178]. Although drugs are administered to reduce symptoms, there is no conventional treatment for endometriosis [179].

Danazol is a pharmacological agent frequently used in pharmacotherapeutic regimens to alleviate the symptoms of endometriosis while having indications in other chronic pathologies such as fibrocystic disease, chronic or persistent refractory immune thrombocytopenia that has not responded to treatment with corticosteroids, or hereditary angioedema [180].

Although used for the abovementioned indications, danazol offers promising results as an anti-ageing agent, maintaining telomeres’ integrity. In a prospective study, daily administration of 800 mg of danazol for 24 months increased TL [181]. In the case of a 42-year-old woman with finger clubbing, a family history of early hair greying led to a TL measure lower than the first centile, thus explaining multiorgan involvement. After 18 months of danazol treatment, the TL was reverted to normal [182]. Córdova-Oriz et al. conducted a double-blind study for three months in which patients with low ovarian function were enrolled. The average TL remained consistent during the three months in the case of patients treated with danazol [183].

## 7. Probiotics

Ageing is an inherent biological process characterised by the gradual decline of the physical condition, leading to the impairment of several physiological functions. All these changes were associated with an accumulation of somatic mutations, oxidative stress, chronic inflammation, mitochondria dysfunction, and denaturation of protein structures, closely related to both the TL and the changes in the intestinal microbiota [184,185,186]. It is well known that a varied range of chronic pathologies are capable of generating changes in the intestinal microbiota, inducing oxidative stress and inflammation, and finally leading to the erosion of telomeres [187]. Therefore, it is pertinent to conclude that there is a robust bidirectional link between the integrity of the intestinal microbiota and TL.

At the same time, broad-spectrum antibiotics reduce the variety of intestinal microbiota; in addition to eliminating the target pathogen, they also destroy beneficial microorganisms, causing harmful effects on the host [188,189]. These changes can lead to a decrease in the variety of microorganisms present, alterations in the functional characteristics, and the development of antibiotic-resistant strains. As a result, the host becomes more vulnerable to infections such as those caused by *Clostridium difficile* [190]. This is one of the reasons that led to the attempts of this century to develop new therapeutic options for treating infections, in addition to the increasing bacterial resistance [191].

**Figure 5:**
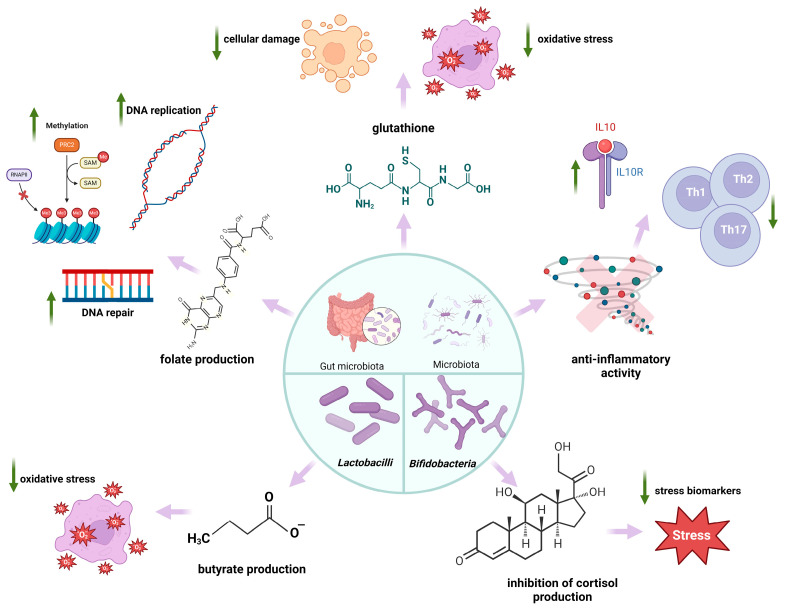
Probiotics on TL dynamics. Legend: IL-10—interleukin 10; IL-10R—interleukin 10 receptor; Th1—Type 1 T helper cells; Th2—Type 2 T helper cells; Th2—Type 2; PRC2—polycomb repressive complex 2; SAMS−adenosyl-methionine; RNAPII—RNA polymerase II; T helper cells; DNA—deoxyribonucleic acid; ↓—decrease; ↑—increase.

Nutritional habits and microbiota profoundly impact TL, supporting the hypothesis of a bidirectional microbiota-TL relationship [192,193]. Probiotics have gained a lot of popularity as food supplements due to their advantages for health, such as restoring the habitat of the gastrointestinal microbiota, competing with harmful pathogens, and improving immune functions [194]. Figure 5 illustrates the main mechanisms supporting the hypothesis of a bidirectional relationship between microbiota and TL and the effects of probiotics on TL maintenance.

Probiotics generate different substances with an antioxidant effect, such as glutathione, butyrate, and folate. *Bifidobacterium* species are studied for their ability to synthesise folate, an essential vitamin for efficient DNA replication, repair, and methylation [195]. At the same time, multiple strains of *Lactobacillus* and *Bifidobacterium* have been shown to exert anti-inflammatory activities by increasing IL-10 levels and decreasing Th1 cytokines. Kwon et al. discovered a combination of probiotics that increases the expression of CD4+forkhead box P3 (FoxP3)+ T-regulatory cells. Therefore, the probiotic combination decreased the sensitivity of both T and B cells and reduced the production of Th1, Th2, and Th17 cytokines without causing apoptosis [196,197]. Furthermore, probiotics have been studied for their potential to reduce cortisol, the body’s main stress hormone [187,198,199]. Table 3 presents some recent studies validating probiotics’ potential to reduce the effects associated with ageing.

Probiotics represent a prospective and easily accessible therapeutic approach to counteracting the effects associated with ageing. However, more studies are required to properly establish the bidirectional relationship between the microbiome and TL.

## 8. Conclusions

Future research directions in this field should focus on large-scale clinical trials to accurately understand the mechanisms involved in hindering telomere erosion mediated by pharmacologically active substances. Moreover, one direction of interest is to investigate the potential of active substances in poly-therapeutic regimens since combining two or more drugs with different mechanisms for inhibition of telomere attrition could bring additional benefits. At the same time, the interactions between the drugs commonly involved in therapy, food, and lifestyle factors should be investigated to obtain more information to guide clinicians in choosing a personalised pharmacotherapeutic regimen.

Additionally, integrating TL measurements into personalised medicine procedures could help to significantly individualise the treatment plan. Thus, physicians and pharmacists could be able to select drugs to treat different pathologies, opting for active substances that maintain genomic stability. However, this can only be achieved with laboratory tests specifically developed to understand telomere dynamics at every single cellular level, not just those measurements that determine the average TL in a cell population. Thus, early identification of patients prone to aberrant shortening of TL at an early stage of the disease may help implement early preventive measures to stop the worsening of the chronic pathology, optimise treatment outcomes, and improve the patient’s long-term quality of life.

In conclusion, this narrative review highlights the prospective role of TL measurements as a reliable biomarker in the treatment of non-communicable diseases. We focused on understanding the molecular mechanisms of those drugs that help inhibit telomere attrition. Investigating the influence of widely used medications on TL offers promising perspectives for developing individualised therapeutic strategies for each patient while offering multiple clinical applications, leading to the efficient management of pathologies and improving health outcomes.

## Figures and Tables

**Table 1:** Antipsychotic therapy and TL in empirical studies.

Methods	Study Groups	Drug Studied	Conclusion	Reference
TL by qPCR	G1: 170 Hispanic patients with SCZ (with antipsychotic therapy) G2: 126 Hispanic healthy controls	Low-risk antipsychotics; atypical antipsychotics (clozapine; olanzapine)	Compared to G2 and G1, using medium and low-risk antipsychotics, G1, with atypical antipsychotics, which cause metabolic syndrome, had severe TL erosion. Olanzapine promotes TL shortening significantly than clozapine.	[133]
Southern blot analysis of mean length of terminal restriction fragment	G1: 34 patients with SCZ that responded well to treatment G2: 35 patients with SCZ that did not respond well to treatment G3: 76 healthy controls	Antipsychotics (analysis conducted regarding treatment adherence)	The subsequent cellular malfunction could contribute to the gradual decline in treatment-resistant SCZ. TL shortening in G2.	[134]
Telomere DNA and PP	G1: antipsychotic treatment-naive SCZ patients G2: control subjects	Antipsychotics (analysis conducted regarding protective effects of drugs after treatment initiation)	Prior to antipsychotic treatment, patients with psychosis had a reduction in telomere DNA content and an elevation in PP.	[135]
TL and mtDNA copy number	G1: 89 patients with 8 weeks on antipsychotic terapy (divided into G1(a)-responders and G1(b)-non-responders) G2: 144 controls	Risperidone	Before risperidone initiation, the TL in G1 was average, but mtDNA was lower than in G2. After risperidone initiation, G1(a), compared to G1(B), had longer TL and lower mtDNA TL and mtDNA could predict response to antipsychotic treatment.	[136]
TL by qPCR	G1: 30 SCZ patients with long-acting injectable antipsychotics G2: 30 SCZ patients with oral atypical antipsychotics	Long-acting injectable antipsychotics; oral atypical antipsychotics	TL might be able to predict how antipsychotic drugs function in SCZ patients.	[137]
			Negative SCZ symptoms are predicted by shorter TL.	
TL by qPCR	1241 SCZ patients	Antipsychotics	Antipsychotic medication had no effect on TL	[138]
	1042 controls			
TL by multiplex qPCR	81 antipsychotic naïve patients 173 SCZ patients 173 healthy controls	Antipsychotics	SCZ patients had longer TL than healthy individuals Patients with non-remitted SCZ exhibited a longer TL than those with remitted SCZ. No effect of antipsychotic medication on TL.	[139]
leukocytes subjected to H_2_O_2_; treated for 7 days with antipsychotics; TL by RT-PCR	Healthy individuals	Aripiprazole; haloperidol; clozapine	Aripiprazole and haloperidol treatment increased TL by 23% and 20% after hydrogen peroxide stimulation	[126]
qPCR for TL and hTERT gene expression, brain neurotrophic factor by ELISA	20 male SCZ patients 20 healthy controls	Antipsychotics	SCZ patients had shorter TL than controls. SCZ patients’ TL increased after antipsychotic treatment. Late-stage patients exhibited a shorter TL than early-stage patients and controls. hTERT gene expression was upregulated during mania and remission.	[140]
TL by qPCR	SCZ patients with early duration of illness (≤5 years) SCZ patients with chronic duration of illness (≥5 years) healthy individuals	Chlorpromazine	Patients with early and chronic psychosis exhibited a considerably prolonged TL in comparison to healthy control subjects. Insignificant correlation between chlorpromazine-equivalent dosages and TL.	[141]

Legend: TL—telomere length; SCZ—schizophrenia; PP—pulse pressure; mtDNA—mitochondrial DNA; qPCR—quantitative polymerase chain reaction; hTERT—human telomerase reverse transcriptase; ELISA—enzyme-linked immunosorbent assay.

**Table 2:** Empirical evidence of the influence of hormone replacement therapy on TL.

Methods	Study Groups	Studied Molecule	Mechanism and Conclusions	Reference
TL by qPCR	G1: 65 women on HT for >5 years G2: 65 women matched in age HT-naive	Oestrogen and progesterone	G1 exhibited greater TL compared to G2. Long-term HT inhibit TL shortening.	[170]
TL by qFISH	Mice	Oestrogen-replacement therapy (ORT)	ORT resulted in elevated levels of TERT gene expression, enhanced telomerase activity, elongated TL, and stimulated ovarian tissue growth. Oestrogen insufficiency or excessive activity can lead to the ageing of ovarian tissue or the development of tumours, respectively, via influencing the remodelling of telomeres through oestrogen control.	[171]
TL by qPCR	1048 women in Sister Study	Oestrogen and progesterone	No association between HT and TL. Reproductive histories that indicate higher levels of naturally occurring oestrogen were linked to shorter TL.	[172]
TL by qPCR	100 newborns from expecting mothers recruited in early pregnancy (tracked prospectively from intrauterine life to early childhood)	Endogenous E3	An elevation in maternal E3 concentration during the initial stages of pregnancy was linked to a 14.42% rise in child TL.	[173]
TRAP for telomerase activity; qPCR for TERT gene expression	G1: female rats G2: female rats with a placebo	21-day release oestrogen formulation	Without oestrogen, the TERT gene’s expression and telomerase activity were decreased. Oestrogen insufficiency leads to a notable reduction in the TL in the adrenal cortex. Oestrogen for 3 weeks restores TERT gene expression, telomerase activity, and cell proliferation.	[174]
TL by qPCR	G1: 333 breast cancer sister-sets G2: 409 unaffected sisters	HT	Shortened TL in unaffected sisters showed a statistically significant correlation with HT-naïve sisters. An inverse relationship was detected between the duration of HT and the TL.	[175]
TL by qPCR	G1: 415 females with IPF G2: 718 males with IPF G3: 204,321 healthy females G4: 174,254 healthy males	Endogenous sex hormones (analysis for the prospective role of HT in telomere maintenance)	Females who experienced early menopause and premature ovarian failure had a greater likelihood of developing IPF. The prevalence of IPF in males was correlated with the levels of bioavailable testosterone in the blood and the stages of the disease. Both males and females showed a correlation between lower levels of sex hormones and shorter TL. Elevated levels of sex hormones provide a protective effect against the development and advancement of IPF, potentially by decelerating the process of TL shortening. Hormonal supplements can potentially delay or prevent the start of diseases in those at risk of telomere-associated IPF and enhance the prognosis of the disease.	[176]
telomerase activity by TRAP; TERT by qPCR	G1: healthy subjects G2: subjects with TERT mutations	androgens; E2	Both drugs increased telomerase activity in G1 in a dose-dependent manner, which was associated with higher amounts of TERT mRNA. Sex hormones activate the expression of TERT by binding to the oestrogen receptor, which can then interact with certain regions in the promoter region of the TERT gene.	[177]

Legend: TL—telomere length; HT—hormone replacement therapy; qPCR—quantitative polymerase chain reaction; TERT—telomerase reverse transcriptase; TRAP—telomerase repeated amplification protocol; E2—estradiol; E3—estriol; IPF—idiopathic pulmonary fibrosis.

**Table 3:** Recent empirical evidence on TL maintenance by probiotics.

Methods	Study Groups	Studied Molecule	Mechanism and Conclusion	Reference
TL and mtDNA by qPCR	G1: hisex brown hens (experimental group) G: hisex brown hens (control group)	0.1% probiotic supplementation (*Bacillus subtilis*)	No significant effect on TL A DNA-protective activity, an inhibition in mtDNA damage by oxidative stress reduction	[200]
TL by qFISH	G1: 16 healthy individuals (experimental group) G2: 31 individuals (control group)	Nutraceutical supplementation (a mixture of various probiotics and vitamins)	The TL measures in G1 were 844 and 953 bases greater than those in G2. Positive impacts on TL by decreasing oxidative stress and inflammation.	[201]
TL by qPCR; AMPK expression	Rats	D-galactose to induce ageing symptoms + groups treated with a statin, *L. reuteri*, *L. fermentum*, *L. plantarum*	Statin, *L. plantarum*, *L. fermentum*, and *L. reuteri* substantially decreased TL shortening and elevated AMPK subunit-α1 expression. Statin and *L. fermentum* treatment significantly reduced plasma lipid peroxidation compared to control. Rats given *L. plantarum* showed higher levels of AMPK subunit-α2 compared to control. *Lactobacillus* probiotics were shown to have strain-dependent efficacy in alleviating age-related impairment.	[202]
TL by RT-PCR	7 groups with 8 mice per group	Milk, yoghurt, *S. thermophilus* metabolites and *L. rhamnosus* metabolites	The yoghurt and *S. thermophilus* group had much longer TL in leucocytes and liver. When t-BHP-challenged HepG2 cells were exposed to digested yoghurt and *S. thermophilus*, the senescence index was reduced, and the TL was increased compared to the control. The yoghurt and metabolites of *S. thermophilus* exhibited antioxidative properties, but the milk and metabolites of *L. rhamnosus* had minimal impact on TL and oxidative stress.	[203]
DNA microarrays for ageing gene expression; HPLC, ELISA, PCR for inflammation markers	G1: mice (experimental group) G2: mice (control group)	*B. lactis* (LKM512)	G1 presented a decrease in the expression of genes linked with ageing and inflammation. Gene expression levels in G1 were similar to those in 10-month-old mice that were not treated (considered younger). G1 had a longer lifespan due to reduced chronic, low-level inflammation in the colon.	[204]
Oxidative stress parameters	Mice	Fermented milk (*L. bulgaricus*, *L. casei*, *S. thermophillus*)	The administration of fermented milk enriched with probiotics for a duration of two weeks resulted in enhanced behaviour, including increased muscular strength, exploratory activity, and reduced anxiety-like behaviour, in addition to better redox status and functionality of peritoneal immune cells in elderly mice.	[205]

Legend: TL—telomere length; mtDNA—mitochondrial DNA; qPCR—quantitative polymerase chain reaction; RT-PCR—real-time polymerase chain reaction; qFISH—quantitative fluorescent in situ hybridisation; AMPK—adenosine monophosphate kinase; HPLC—high-performance liquid chromatography; ELISA—enzyme-linked immunosorbent assay; t-BHP—tert butyl hydroperoxide.

## References

[1] Revy P., Kannengiesser C., Bertuch A.A. (2023). Genetics of Human Telomere Biology Disorders. Nat. Rev. Genet..

[2] Tsatsakis A., Renieri E., Tsoukalas D., Buga A., Sarandi E., Vakonaki E., Fragkiadaki P., Alegakis A., Nikitovic D., Calina D. (2023). A Novel Nutraceutical Formulation Increases Telomere Length and Activates Telomerase Activity in Middle-aged Rats. Mol. Med. Rep..

[3] Tsoukalas D., Buga A., Docea A., Sarandi E., Mitrut R., Renieri E., Spandidos D., Rogoveanu I., Cercelaru L., Niculescu M. (2021). Reversal of Brain Aging by Targeting Telomerase: A Nutraceutical Approach. Int. J. Mol. Med..

[4] Rossiello F., Jurk D., Passos J.F., d’Adda di Fagagna F. (2022). Telomere Dysfunction in Ageing and Age-Related Diseases. Nat. Cell Biol..

[5] Renieri E., Vakonaki E., Karzi V., Fragkiadaki P., Tsatsakis A.M. (2021). Telomere Length: Associations with Nutrients and Xenobiotics. Toxicological Risk Assessment and Multi-System Health Impacts from Exposure.

[6] López-Otín C., Blasco M.A., Partridge L., Serrano M., Kroemer G. (2023). Hallmarks of Aging: An Expanding Universe. Cell.

[7] Chakravarti D., LaBella K.A., DePinho R.A. (2021). Telomeres: History, Health, and Hallmarks of Aging. Cell.

[8] Bhattacharyya J., Mihara K., Bhattacharjee D., Mukherjee M. (2017). Telomere Length as a Potential Biomarker of Coronary Artery Disease. Indian J. Med. Res..

[9] Cheng F., Carroll L., Joglekar M.V., Januszewski A.S., Wong K.K., Hardikar A.A., Jenkins A.J., Ma R.C.W. (2021). Diabetes, Metabolic Disease, and Telomere Length. Lancet Diabetes Endocrinol..

[10] Fragkiadaki P., Nikitovic D., Kalliantasi K., Sarandi E., Thanasoula M., Stivaktakis P., Nepka C., Spandidos D., Theodoros T., Tsatsakis A. (2019). Telomere Length and Telomerase Activity in Osteoporosis and Osteoarthritis (Review). Exp. Ther. Med..

[11] Rodríguez-Fernández B., Gispert J.D., Guigo R., Navarro A., Vilor-Tejedor N., Crous-Bou M. (2022). Genetically Predicted Telomere Length and Its Relationship with Neurodegenerative Diseases and Life Expectancy. Comput. Struct. Biotechnol. J..

[12] Pousa P.A., Souza R.M., Melo P.H.M., Correa B.H.M., Mendonça T.S.C., Simões-e-Silva A.C., Miranda D.M. (2021). Telomere Shortening and Psychiatric Disorders: A Systematic Review. Cells.

[13] Vasilopoulos E., Fragkiadaki P., Kalliora C., Fragou D., Docea A., Vakonaki E., Tsoukalas D., Calina D., Buga A., Georgiadis G. (2019). The Association of Female and Male Infertility with Telomere Length (Review). Int. J. Mol. Med..

[14] Tsatsakis A., Oikonomopoulou T., Nikolouzakis T., Vakonaki E., Tzatzarakis M., Flamourakis M., Renieri E., Fragkiadaki P., Iliaki E., Bachlitzanaki M. (2023). Role of Telomere Length in Human Carcinogenesis (Review). Int. J. Oncol..

[15] Unni E. (2023). Medicine Use in Chronic Diseases. Pharmacy.

[16] Tenchov R., Sasso J.M., Wang X., Zhou Q.A. (2024). Aging Hallmarks and Progression and Age-Related Diseases: A Landscape View of Research Advancement. ACS Chem. Neurosci..

[17] Dominguez L., Veronese N., Barbagallo M. (2024). Magnesium and the Hallmarks of Aging. Nutrients.

[18] Shammas M.A. (2011). Telomeres, Lifestyle, Cancer, and Aging. Curr. Opin. Clin. Nutr. Metab. Care.

[19] Guo J., Huang X., Dou L., Yan M., Shen T., Tang W., Li J. (2022). Aging and Aging-Related Diseases: From Molecular Mechanisms to Interventions and Treatments. Signal Transduct. Target. Ther..

[20] Mylonas A., O’Loghlen A. (2022). Cellular Senescence and Ageing: Mechanisms and Interventions. Front. Aging.

[21] Li Y., Tian X., Luo J., Bao T., Wang S., Wu X. (2024). Molecular Mechanisms of Aging and Anti-Aging Strategies. Cell Commun. Signal..

[22] Mc Auley M.T., Guimera A.M., Hodgson D., Mcdonald N., Mooney K.M., Morgan A.E., Proctor C.J. (2017). Modelling the Molecular Mechanisms of Aging. Biosci. Rep..

[23] Wang K., Liu H., Hu Q., Wang L., Liu J., Zheng Z., Zhang W., Ren J., Zhu F., Liu G.-H. (2022). Epigenetic Regulation of Aging: Implications for Interventions of Aging and Diseases. Signal Transduct. Target. Ther..

[24] Pyo I.S., Yun S., Yoon Y.E., Choi J.-W., Lee S.-J. (2020). Mechanisms of Aging and the Preventive Effects of Resveratrol on Age-Related Diseases. Molecules.

[25] Gopenath T.S., Shreshtha S., Basalingappa K.M. (2022). Telomerase Reactivation for Anti-Aging. Anti-Aging Drug Discovery on the Basis of Hallmarks of Aging.

[26] Razgonova M., Zakharenko A., Golokhvast K., Thanasoula M., Sarandi E., Nikolouzakis K., Fragkiadaki P., Tsoukalas D., Spandidos D., Tsatsakis A. (2020). Telomerase and Telomeres in Aging Theory and Chronographic Aging Theory (Review). Mol. Med. Rep..

[27] Tsoukalas D., Fragkiadaki P., Docea A., Alegakis A., Sarandi E., Thanasoula M., Spandidos D., Tsatsakis A., Razgonova M., Calina D. (2019). Discovery of Potent Telomerase Activators: Unfolding New Therapeutic and Anti-Aging Perspectives. Mol. Med. Rep..

[28] Hou K., Yu Y., Li D., Zhang Y., Zhang K., Tong J., Yang K., Jia S. (2022). Alternative Lengthening of Telomeres and Mediated Telomere Synthesis. Cancers.

[29] Silva B., Arora R., Azzalin C.M. (2022). The Alternative Lengthening of Telomeres Mechanism Jeopardizes Telomere Integrity If Not Properly Restricted. Proc. Natl. Acad. Sci. USA.

[30] Di Micco R., Krizhanovsky V., Baker D., d’Adda di Fagagna F. (2021). Cellular Senescence in Ageing: From Mechanisms to Therapeutic Opportunities. Nat. Rev. Mol. Cell Biol..

[31] Zhang L., Pitcher L.E., Yousefzadeh M.J., Niedernhofer L.J., Robbins P.D., Zhu Y. (2022). Cellular Senescence: A Key Therapeutic Target in Aging and Diseases. J. Clin. Investig..

[32] Kumari R., Jat P. (2021). Mechanisms of Cellular Senescence: Cell Cycle Arrest and Senescence Associated Secretory Phenotype. Front. Cell Dev. Biol..

[33] Roth G.A., Mensah G.A., Johnson C.O., Addolorato G., Ammirati E., Baddour L.M., Barengo N.C., Beaton A.Z., Benjamin E.J., Benziger C.P. (2020). Global Burden of Cardiovascular Diseases and Risk Factors, 1990–2019. J. Am. Coll. Cardiol..

[34] Joseph P., Leong D., McKee M., Anand S.S., Schwalm J.-D., Teo K., Mente A., Yusuf S. (2017). Reducing the Global Burden of Cardiovascular Disease, Part 1. Circ. Res..

[35] Huang Y.-C., Wang C.-Y. (2021). Telomere Attrition and Clonal Hematopoiesis of Indeterminate Potential in Cardiovascular Disease. Int. J. Mol. Sci..

[36] Hoffmann J., Richardson G., Haendeler J., Altschmied J., Andrés V., Spyridopoulos I. (2021). Telomerase as a Therapeutic Target in Cardiovascular Disease. Arterioscler. Thromb. Vasc. Biol..

[37] Xu C., Wang Z., Su X., Da M., Yang Z., Duan W., Mo X. (2020). Association between Leucocyte Telomere Length and Cardiovascular Disease in a Large General Population in the United States. Sci. Rep..

[38] Haycock P.C., Heydon E.E., Kaptoge S., Butterworth A.S., Thompson A., Willeit P. (2014). Leucocyte Telomere Length and Risk of Cardiovascular Disease: Systematic Review and Meta-Analysis. BMJ.

[39] Yeh J.-K., Wang C.-Y. (2016). Telomeres and Telomerase in Cardiovascular Diseases. Genes.

[40] Boccardi V., Paolisso G. (2014). The Association between Statins and Telomere Shortening. Clin. Lipidol..

[41] Zhuang X.-D., Liao L.-Z., Guo Y., Li Y., Liao X.-X., Hu X., Du Z.-M. (2015). Rationale and Design of RETAIN Study: Rosuvastatin Effect on Telomere–Telomerase System in Acute Coronary Syndrome Patients Undergoing Percutaneous Coronary Intervention. Int. J. Cardiol..

[42] Martynowicz H., Gać P., Kornafel-Flak O., Filipów S., Łaczmański Ł., Sobieszczańska M., Mazur G., Porȩba R. (2020). The Relationship Between the Effectiveness of Blood Pressure Control and Telomerase Reverse Transcriptase Concentration, Adipose Tissue Hormone Concentration and Endothelium Function in Hypertensives. Hear Lung Circ..

[43] Vasan R.S., Demissie S., Kimura M., Cupples L.A., Rifai N., White C., Wang T.J., Gardner J.P., Cao X., Benjamin E.J. (2008). Association of Leukocyte Telomere Length with Circulating Biomarkers of the Renin-Angiotensin-Aldosterone System. Circulation.

[44] Nemtsova V., Bondar T., Shalimova A. (2020). Effect of Achieving Blood Pressure Targets on the Relative Telomere Length in Hypertensive Patients with and without Type 2 Diabetes Mellitus. Arter. Hypertens..

[45] Harvey A., Montezano A.C., Touyz R.M. (2015). Vascular Biology of Ageing—Implications in Hypertension. J. Mol. Cell. Cardiol..

[46] Dayar E., Pechanova O. (2022). Targeted Strategy in Lipid-Lowering Therapy. Biomedicines.

[47] Pinal-Fernandez I., Casal-Dominguez M., Mammen A.L. (2018). Statins: Pros and Cons. Med. Clin..

[48] Morofuji Y., Nakagawa S., Ujifuku K., Fujimoto T., Otsuka K., Niwa M., Tsutsumi K. (2022). Beyond Lipid-Lowering: Effects of Statins on Cardiovascular and Cerebrovascular Diseases and Cancer. Pharmaceuticals.

[49] Choudhary A., Rawat U., Kumar P., Mittal P. (2023). Pleotropic Effects of Statins: The Dilemma of Wider Utilization of Statin. Egypt. Hear J..

[50] Hu G., Long A., Wang J., Wang X. (2022). Effects of Oral Atorvastatin on Inflammatory Markers and Postoperative Delirium in Elderly Patients with Hip Fracture Surgery. Farmacia.

[51] Nose D., Shiga Y., Takahashi R., Yamamoto Y., Suematsu Y., Kuwano T., Sugihara M., Kanda M., Tahara H., Miura S. (2023). Association Between Telomere G-Tail Length and Coronary Artery Disease or Statin Treatment in Patients with Cardiovascular Risks—A Cross-Sectional Study—. Circ. Rep..

[52] Gorabi A.M., Kiaie N., Hajighasemi S., Banach M., Penson P.E., Jamialahmadi T., Sahebkar A. (2019). Statin-Induced Nitric Oxide Signaling: Mechanisms and Therapeutic Implications. J. Clin. Med..

[53] Mansouri A., Reiner Ž., Ruscica M., Tedeschi-Reiner E., Radbakhsh S., Bagheri Ekta M., Sahebkar A. (2022). Antioxidant Effects of Statins by Modulating Nrf2 and Nrf2/HO-1 Signaling in Different Diseases. J. Clin. Med..

[54] Jang H.J., Hong E.M., Kim M., Kim J.H., Jang J., Park S.W., Byun H.W., Koh D.H., Choi M.H., Kae S.H. (2016). Simvastatin Induces Heme Oxygenase-1 via NF-E2-Related Factor 2 (Nrf2) Activation through ERK and PI3K/Akt Pathway in Colon Cancer. Oncotarget.

[55] Sugimoto M., Ko R., Goshima H., Koike A., Shibano M., Fujimori K. (2021). Formononetin Attenuates H2O2-Induced Cell Death through Decreasing ROS Level by PI3K/Akt-Nrf2-Activated Antioxidant Gene Expression and Suppressing MAPK-Regulated Apoptosis in Neuronal SH-SY5Y Cells. Neurotoxicology.

[56] Olivieri F., Mazzanti I., Abbatecola M.A., Recchioni R., Marcheselli F., Procopio D.A., Antonicelli R. (2012). Telomere/Telomerase System: A New Target of Statins Pleiotropic Effect?. Curr. Vasc. Pharmacol..

[57] Zaky M.Y., Fan C., Zhang H., Sun X.-F. (2023). Unraveling the Anticancer Potential of Statins: Mechanisms and Clinical Significance. Cancers.

[58] Tan X.W., Fong A.Y.Y. (2023). Telomere and Telomerase Biology in Cardiovascular Disease: A State-of-the-Art Review and Outlook. J. Asian Pacific Soc. Cardiol..

[59] Collins R., Reith C., Emberson J., Armitage J., Baigent C., Blackwell L., Blumenthal R., Danesh J., Smith G.D., DeMets D. (2016). Interpretation of the Evidence for the Efficacy and Safety of Statin Therapy. Lancet.

[60] Cheng J. (2014). Statin Therapy Decreased the Recurrence Frequency of Atrial Fibrillation after Electrical Cardioversion: A Meta-Analysis. Med. Sci. Monit..

[61] Tiwari A. (2007). Statins and Telomere Length: Risk Assessment and Management for Coronary Heart Disease. Expert Opin. Ther. Targets.

[62] Bennaceur K., Atwill M., Al Zhrany N., Hoffmann J., Keavney B., Breault D., Richardson G., von Zglinicki T., Saretzki G., Spyridopoulos I. (2014). Atorvastatin Induces T Cell Proliferation by a Telomerase Reverse Transcriptase (TERT) Mediated Mechanism. Atherosclerosis.

[63] Strazhesko I.D., Tkacheva O.N., Akasheva D.U., Dudinskaya E.N., Plokhova E.V., Pykhtina V.S., Kruglikova A.S., Kokshagina N.V., Sharashkina N.V., Agaltsov M.V. (2016). Atorvastatin Therapy Modulates Telomerase Activity in Patients Free of Atherosclerotic Cardiovascular Diseases. Front. Pharmacol..

[64] McKeever R., Hamilton R. (2022). Calcium Channel Blockers.

[65] Elliott W.J., Ram C.V.S. (2011). Calcium Channel Blockers. J. Clin. Hypertens..

[66] Zhu J., Chen N., Zhou M., Guo J., Zhu C., Zhou J., Ma M., He L. (2022). Calcium Channel Blockers versus Other Classes of Drugs for Hypertension. Cochrane Database Syst. Rev..

[67] Haller H. (2008). Effective Management of Hypertension with Dihydropyridine Calcium Channel Blocker-Based Combination Therapy in Patients at High Cardiovascular Risk. Int. J. Clin. Pract..

[68] Hayashi T., Yamaguchi T., Sakakibara Y., Taguchi K., Maeda M., Kuzuya M., Hattori Y. (2014). ENOS-Dependent Antisenscence Effect of a Calcium Channel Blocker in Human Endothelial Cells. PLoS ONE.

[69] Godfraind T. (2006). Calcium-Channel Modulators for Cardiovascular Disease. Expert Opin. Emerg. Drugs.

[70] Tang B., Li X., Wang Y., Sjölander A., Johnell K., Thambisetty M., Ferrucci L., Reynolds C.A., Finkel D., Jylhävä J. (2023). Longitudinal Associations between Use of Antihypertensive, Antidiabetic, and Lipid-Lowering Medications and Biological Aging. GeroScience.

[71] Zhang S.Y., Li R.X., Yang Y.Y., Chen Y., Yang S.J., Li J., Fu L., Hui R.T., Zhang W.L. (2019). P1693The Longitudinal Associations between Telomere Attrition and the Effects of Blood Pressure Lowering and Antihypertensive Treatment. Eur. Heart J..

[72] Ramalingam L., Menikdiwela K., LeMieux M., Dufour J.M., Kaur G., Kalupahana N., Moustaid-Moussa N. (2017). The Renin Angiotensin System, Oxidative Stress and Mitochondrial Function in Obesity and Insulin Resistance. Biochim. Biophys. Acta Mol. Basis Dis..

[73] Maranduca M.A., Cosovanu M.A., Clim A., Pinzariu A.C., Filip N., Drochioi I.C., Vlasceanu V.I., Timofte D.V., Nemteanu R., Plesa A. (2023). The Renin-Angiotensin System: The Challenge behind Autoimmune Dermatological Diseases. Diagnostics.

[74] Yi W., Chen F., Zhang H., Tang P., Yuan M., Wen J., Wang S., Cai Z. (2022). Role of Angiotensin II in Aging. Front. Aging Neurosci..

[75] Yeh J.-K., Lin M.-H., Wang C.-Y. (2019). Telomeres as Therapeutic Targets in Heart Disease. JACC Basic Transl. Sci..

[76] Saavedra J.M. (2021). Angiotensin Receptor Blockers Are Not Just for Hypertension Anymore. Physiology.

[77] Lee H.W., Kim S., Jo Y., Kim Y., Ye B.S., Yu Y.M. (2023). Neuroprotective Effect of Angiotensin II Receptor Blockers on the Risk of Incident Alzheimer’s Disease: A Nationwide Population-Based Cohort Study. Front. Aging Neurosci..

[78] Weinstein J.R., Anderson S. (2010). The Aging Kidney: Physiological Changes. Adv. Chronic Kidney Dis..

[79] Aoulad Fares D., Wiegel R.E., Eggink A.J., van Meurs J.B.J., Willemsen S.P., Danser A.H.J., Steegers-Theunissen R.P.M. (2023). First-Trimester Maternal Renin-Angiotensin-Aldosterone System Activation and the Association with Maternal Telomere Length after Natural and IVF/ICSI Conceived Pregnancies: The Rotterdam Periconception Cohort. Hypertens. Pregnancy.

[80] Baumann M., Bartholome R., Peutz-Kootstra C.J., Smits J.F.M., Struijker-Boudier H.A.J. (2008). Sustained Tubulo-Interstitial Protection in SHRs by Transient Losartan Treatment: An Effect of Decelerated Aging?. Am. J. Hypertens..

[81] Feng X., Wang L., Li Y. (2011). Change of Telomere Length in Angiotensin Ii-Induced Human Glomerular Mesangial Cell Senescence and the Protective Role of Losartan. Mol. Med. Rep..

[82] Fyhrquist F., Tiitu A., Saijonmaa O., Forsblom C., Groop P.-H. (2010). Telomere Length and Progression of Diabetic Nephropathy in Patients with Type 1 Diabetes. J. Intern. Med..

[83] Ancion A., Tridetti J., Nguyen Trung M.-L., Oury C., Lancellotti P. (2019). A Review of the Role of Bradykinin and Nitric Oxide in the Cardioprotective Action of Angiotensin-Converting Enzyme Inhibitors: Focus on Perindopril. Cardiol. Ther..

[84] Obtułowicz K., Góralska J., Bogdali A., Dyga W., Obtułowicz A., Myszkowska D., Ziemianin M., Gruca A., Solnica B., Czarnobilska E. (2020). Bradykinin and Oxidative Stress in Patients with Hereditary Angioedema Due to C1 Inhibitor Deficiency. Polish Arch. Intern. Med..

[85] Donnini S., Terzuoli E., Ziche M., Morbidelli L. (2010). Sulfhydryl Angiotensin-Converting Enzyme Inhibitor Promotes Endothelial Cell Survival through Nitric-Oxide Synthase, Fibroblast Growth Factor-2, and Telomerase Cross-Talk. J. Pharmacol. Exp. Ther..

[86] De Vries N., Prestes P., Raipuria M., Byars S., Allen A., Harrap S., Charchar F. (2018). A15812 ANGIOTENSIN CONVERTING ENZYME INHIBITORS EPIGENETICALLY ATENUATE TELOMERE SHORTENING. J. Hypertens..

[87] Akinnibosun O., Prestes P., De Vries N., Raipuria M., Byars S., Allen A., Harrap S., Charchar F. (2023). Investigation of Telomere Involvement in the Legacy Effect of Angiotensin Converting Enzyme Inhibitors in Spontaneously Hypertensive Rats. Hear Lung Circ..

[88] WHO Diabetes. https://www.who.int/news-room/fact-sheets/detail/diabetes.

[89] Farmaki P., Damaskos C., Garmpis N., Garmpi A., Savvanis S., Diamantis E. (2021). Complications of the Type 2 Diabetes Mellitus. Curr. Cardiol. Rev..

[90] Tilinca M.C., Antal C., Sălcudean A., Abălașei B.L., Fărcaș R.M., Groșan A. (2023). New Directions in Pharmacological Treatment with SGLT-2 Inhibitor Molecules in the Light of Current Guidelines for Diabetes Mellitus, Heart Failure and Kidney Disease. Farmacia.

[91] Abdulsaied R.A., Jabbar A.S., Akar T.K. (2022). Disorders of Pulmonary Function in Type 2 Diabetes Mellitus Patients with Different Types of Oral Hypoglycemic Medications: Metformin, Metformin Plus Thiazolidinedione, and Metformin Plus Sulfonylurea. Farmacia.

[92] Cheng F., Luk A.O., Shi M., Huang C., Jiang G., Yang A., Wu H., Lim C.K.P., Tam C.H.T., Fan B. (2022). Shortened Leukocyte Telomere Length Is Associated with Glycemic Progression in Type 2 Diabetes: A Prospective and Mendelian Randomization Analysis. Diabetes Care.

[93] Chaithanya V., Kumar J., Leela K.V., Murugesan R., Angelin M., Satheesan A. (2023). Impact of Telomere Attrition on Diabetes Mellitus and Its Complications. Diabetes Epidemiol. Manag..

[94] Qin B. (2023). Can Antidiabetic Medications Affect Telomere Length in Patients with Type 2 Diabetes? A Mini-Review. Diabetes Metab. Syndr. Obes..

[95] Wang J., Dong X., Cao L., Sun Y., Qiu Y., Zhang Y., Cao R., Covasa M., Zhong L. (2016). Association between Telomere Length and Diabetes Mellitus: A Meta-Analysis. J. Int. Med. Res..

[96] Baltzis D., Meimeti E., Grammatikopoulou M., Roustit M., Mavrogonatou E., Kletsas D., Efraimidou S., Manes C., Nikolouzakis T., Tsiaoussis J. (2018). Assessment of Telomerase Activity in Leukocytes of Type 2 Diabetes Mellitus Patients Having or Not Foot Ulcer: Possible Correlation with Other Clinical Parameters. Exp. Ther. Med..

[97] Volpe C.M.O., Villar-Delfino P.H., dos Anjos P.M.F., Nogueira-Machado J.A. (2018). Cellular Death, Reactive Oxygen Species (ROS) and Diabetic Complications. Cell Death Dis..

[98] Yu T., Jhun B.S., Yoon Y. (2011). High-Glucose Stimulation Increases Reactive Oxygen Species Production Through the Calcium and Mitogen-Activated Protein Kinase-Mediated Activation of Mitochondrial Fission. Antioxid. Redox Signal..

[99] Cuevas Diaz P., Nicolini H., Nolasco-Rosales G.A., Juarez Rojop I., Tovilla-Zarate C.A., Rodriguez Sanchez E., Genis-Mendoza A.D. (2023). Telomere Shortening in Three Diabetes Mellitus Types in a Mexican Sample. Biomedicines.

[100] Tamura Y., Izumiyama-Shimomura N., Kimbara Y., Nakamura K., Ishikawa N., Aida J., Chiba Y., Mori S., Arai T., Aizawa T. (2014). β-Cell Telomere Attrition in Diabetes: Inverse Correlation Between HbA1c and Telomere Length. J. Clin. Endocrinol. Metab..

[101] González P., Lozano P., Ros G., Solano F. (2023). Hyperglycemia and Oxidative Stress: An Integral, Updated and Critical Overview of Their Metabolic Interconnections. Int. J. Mol. Sci..

[102] Monickaraj F., Aravind S., Gokulakrishnan K., Sathishkumar C., Prabu P., Prabu D., Mohan V., Balasubramanyam M. (2012). Accelerated Aging as Evidenced by Increased Telomere Shortening and Mitochondrial DNA Depletion in Patients with Type 2 Diabetes. Mol. Cell. Biochem..

[103] Wu Y., Cui W., Zhang D., Wu W., Yang Z. (2017). The Shortening of Leukocyte Telomere Length Relates to DNA Hypermethylation of LINE-1 in Type 2 Diabetes Mellitus. Oncotarget.

[104] Ojeda-Rodriguez A., Alcala-Diaz J.F., Rangel-Zuñiga O.A., Arenas-de Larriva A.P., Gutierrez-Mariscal F.M., Torres-Peña J.D., Mora-Ortiz M., Romero-Cabrera J.L., Luque R.M., Ordovas J.M. (2024). Telomere Maintenance Is Associated with Type 2 Diabetes Remission in Response to a Long-Term Dietary Intervention without Non-Weight Loss in Patients with Coronary Heart Disease: From the CORDIOPREV Randomized Controlled Trial. Antioxidants.

[105] Zeng J., Liu H., Ping F., Li W., Li Y. (2019). Insulin Treatment Affects Leukocyte Telomere Length in Patients with Type 2 Diabetes: 6-Year Longitudinal Study. J. Diabetes Complicat..

[106] Mangge H., Herrmann M., Almer G., Zelzer S., Moeller R., Horejsi R., Renner W. (2020). Telomere Shortening Associates with Elevated Insulin and Nuchal Fat Accumulation. Sci. Rep..

[107] Baker C., Retzik-Stahr C., Singh V., Plomondon R., Anderson V., Rasouli N. (2021). Should Metformin Remain the First-Line Therapy for Treatment of Type 2 Diabetes?. Ther. Adv. Endocrinol. Metab..

[108] Apostu A., Buzatu R., Cabuta M., Macasoi I., Dinu S., Iftode A., Mânea H.C., Gaita D.I., Chiriac S.D. (2023). In Vitro Assessment of the Potential Cytotoxic Effect of Metformin on Colorectal Cancer Cells. Farmacia.

[109] Chen S., Gan D., Lin S., Zhong Y., Chen M., Zou X., Shao Z., Xiao G. (2022). Metformin in Aging and Aging-Related Diseases: Clinical Applications and Relevant Mechanisms. Theranostics.

[110] Son D.-H., Park W.-J., Lee Y.-J. (2019). Recent Advances in Anti-Aging Medicine. Korean J. Fam. Med..

[111] Sevim C., Taghizadehghalehjoughi A., Kara M., Nosyrev A.E., Nițulescu G.M., Margină D., Tsatsakis A. (2024). Investigation of the Effects of Metformin on the miR21/PTEN/Akt Pathway in HT-29 Human Colorectal Adenocarcinoma Cell and HUVEC Co-Culture. Farmacia.

[112] Mohammed I., Hollenberg M.D., Ding H., Triggle C.R. (2021). A Critical Review of the Evidence That Metformin Is a Putative Anti-Aging Drug That Enhances Healthspan and Extends Lifespan. Front. Endocrinol..

[113] Chrienova Z., Nepovimova E., Kuca K. (2021). The Role of MTOR in Age-Related Diseases. J. Enzyme Inhib. Med. Chem..

[114] Ma T., Tian X., Zhang B., Li M., Wang Y., Yang C., Wu J., Wei X., Qu Q., Yu Y. (2022). Low-Dose Metformin Targets the Lysosomal AMPK Pathway through PEN2. Nature.

[115] Foretz M., Guigas B., Viollet B. (2023). Metformin: Update on Mechanisms of Action and Repurposing Potential. Nat. Rev. Endocrinol..

[116] Baur J.A., Birnbaum M.J. (2014). Control of Gluconeogenesis by Metformin: Does Redox Trump Energy Charge?. Cell Metab..

[117] Luo S., Wong I.C.K., Chui C.S.L., Zheng J., Huang Y., Schooling C.M., Yeung S.L.A. (2023). Effects of Putative Metformin Targets on Phenotypic Age and Leukocyte Telomere Length: A Mendelian Randomisation Study Using Data from the UK Biobank. Lancet Heal. Longev..

[118] Yang J., Liu H.-C., Zhang J.-Q., Zou J.-Y., Zhang X., Chen W.-M., Gu Y., Hong H. (2023). The Effect of Metformin on Senescence of T Lymphocytes. Immun. Ageing.

[119] Vigili de Kreutzenberg S., Ceolotto G., Cattelan A., Pagnin E., Mazzucato M., Garagnani P., Borelli V., Bacalini M.G., Franceschi C., Fadini G.P. (2015). Metformin Improves Putative Longevity Effectors in Peripheral Mononuclear Cells from Subjects with Prediabetes. A Randomized Controlled Trial. Nutr. Metab. Cardiovasc. Dis..

[120] Yang Y., Liu Y., Wang Y., Chao Y., Zhang J., Jia Y., Tie J., Hu D. (2022). Regulation of SIRT1 and Its Roles in Inflammation. Front. Immunol..

[121] Florentin M., Kostapanos M.S., Papazafiropoulou A.K. (2022). Role of Dipeptidyl Peptidase 4 Inhibitors in the New Era of Antidiabetic Treatment. World J. Diabetes.

[122] Saini K., Sharma S., Khan Y. (2023). DPP-4 Inhibitors for Treating T2DM—Hype or Hope? An Analysis Based on the Current Literature. Front. Mol. Biosci..

[123] Dudinskaya E., Matchekhina L., Strazhesko I., Tkacheva O. (2021). Combined Vildagliptin + Metformin Therapy Can Increase Telomerase Activity in Patients with Type 2 Diabetes. Endocr. Abstr..

[124] Vakonaki E., Tsiminikaki K., Plaitis S., Fragkiadaki P., Tsoukalas D., Katsikantami I., Vaki G., Tzatzarakis M., Spandidos D., Tsatsakis A. (2018). Common Mental Disorders and Association with Telomere Length (Review). Biomed. Rep..

[125] Yonezawa K., Kanegae S., Ozawa H. (2021). Antipsychotics/Neuroleptics: Pharmacology and Biochemistry. NeuroPsychopharmacotherapy.

[126] Polho G., Cardillo G., Kerr D., Chile T., Gattaz W., Forlenza O., Brentani H., De-Paula V. (2022). Antipsychotics Preserve Telomere Length in Peripheral Blood Mononuclear Cells after Acute Oxidative Stress Injury. Neural Regen. Res..

[127] Wang D., Li H., Du X., Zhou J., Yuan L., Ren H., Yang X., Zhang G., Chen X. (2020). Circulating Brain-Derived Neurotrophic Factor, Antioxidant Enzymes Activities, and Mitochondrial DNA in Bipolar Disorder: An Exploratory Report. Front. Psychiatry.

[128] Gurevich A., Guller V., Berner Y.N., Tal S. (2012). Are Atypical Antipsychotics Safer than Typical Antipsychotics for Treating Behavioral and Psychological Symptoms of Dementia?. J. Nutr. Health Aging.

[129] Jia T., Len X., Pi Z., Hong Z., Feng J., Ma C. (2022). Effect of Aripiprazole Combined with Olanzapine on the Clinical Efficacy of Schizophrenia. Farmacia.

[130] Caruso G., Grasso M., Fidilio A., Tascedda F., Drago F., Caraci F. (2020). Antioxidant Properties of Second-Generation Antipsychotics: Focus on Microglia. Pharmaceuticals.

[131] Blandino G., Fiorani M., Canonico B., De Matteis R., Guidarelli A., Montanari M., Buffi G., Coppo L., Arnér E.S.J., Cantoni O. (2023). Clozapine Suppresses NADPH Oxidase Activation, Counteracts Cytosolic H2O2, and Triggers Early Onset Mitochondrial Dysfunction during Adipogenesis of Human Liposarcoma SW872 Cells. Redox Biol..

[132] Ben-Azu B., del Re E.C., VanderZwaag J., Carrier M., Keshavan M., Khakpour M., Tremblay M.-È. (2023). Emerging Epigenetic Dynamics in Gut-Microglia Brain Axis: Experimental and Clinical Implications for Accelerated Brain Aging in Schizophrenia. Front. Cell. Neurosci..

[133] Monroy-Jaramillo N., Rodríguez-Agudelo Y., Aviña-Cervantes L.C., Roberts D.L., Velligan D.I., Walss-Bass C. (2017). Leukocyte Telomere Length in Hispanic Schizophrenia Patients under Treatment with Olanzapine. J. Psychiatr. Res..

[134] Yu W.-Y., Chang H.-W., Lin C.-H., Cho C.-L. (2008). Short Telomeres in Patients with Chronic Schizophrenia Who Show a Poor Response to Treatment. J. Psychiatry Neurosci..

[135] Fernandez-Egea E., Bernardo M., Heaphy C.M., Griffith J.K., Parellada E., Esmatjes E., Conget I., Nguyen L., George V., Stoppler H. (2009). Telomere Length and Pulse Pressure in Newly Diagnosed, Antipsychotic-Naive Patients with Nonaffective Psychosis. Schizophr. Bull..

[136] Li Z., Hu M., Zong X., He Y., Wang D., Dai L., Dong M., Zhou J., Cao H., Lv L. (2015). Association of Telomere Length and Mitochondrial DNA Copy Number with Risperidone Treatment Response in First-Episode Antipsychotic-Naïve Schizophrenia. Sci. Rep..

[137] Pippal N., Halder S., Srivastava S., Kar R., Gupta R., Anthonio A.E. (2022). Correlation between Telomere Length and Efficacy of Oral and Long-Acting Injectable Antipsychotics on Severity and Cognitive Impairment of Schizophrenia. Int. J. Psychiatry Clin. Pract..

[138] Zhang Y., Hishimoto A., Otsuka I., Watanabe Y., Numata S., Yamamori H., Boku S., Horai T., Someya T., Ohmori T. (2018). Longer Telomeres in Elderly Schizophrenia Are Associated with Long-Term Hospitalization in the Japanese Population. J. Psychiatr. Res..

[139] Maurya P.K., Rizzo L.B., Xavier G., Tempaku P.F., Ota V.K., Santoro M.L., Spíndola L.M., Moretti P.S., Mazzotti D.R., Gadelha A. (2018). Leukocyte Telomere Length Variation in Different Stages of Schizophrenia. J. Psychiatr. Res..

[140] Köse Çinar R. (2017). Telomere Length and HTERT in Mania and Subsequent Remission. Rev. Bras. Psiquiatr..

[141] Cui Y., Prabhu V.V., Nguyen T.B., Devi S.M., Chung Y.-C. (2017). Longer Telomere Length of T Lymphocytes in Patients with Early and Chronic Psychosis. Clin. Psychopharmacol. Neurosci..

[142] Masters A., Pandi-Perumal S.R., Seixas A., Girardin J.L., McFarlane S.I. (2015). Melatonin, the Hormone of Darkness: From Sleep Promotion to Ebola Treatment. Brain Disord. Ther..

[143] Ţincu R.C., Ivan A.S., Cobilinschi C., Ţincu I.F., Macovei R.A. (2022). 5-Hydroxytryptophan Dietary Supplementation in Post-Traumatic Stress Syndrome. Farmacia.

[144] Horodincu L., Solcan C. (2023). Influence of Different Light Spectra on Melatonin Synthesis by the Pineal Gland and Influence on the Immune System in Chickens. Animals.

[145] Poza J.J., Pujol M., Ortega-Albás J.J., Romero O. (2022). Melatonina En Los Trastornos de Sueño. Neurología.

[146] Stancu E., Carata A., Tăerel A.-E. (2015). From the History of Drugs: Oleum Jecoris Aselli, a Long Time Used Remedy. Farmacia.

[147] Sharkey K.M., Fogg L.F., Eastman C.I. (2001). Effects of Melatonin Administration on Daytime Sleep after Simulated Night Shift Work. J. Sleep Res..

[148] Ogundele M.O., Yemula C. (2022). Management of Sleep Disorders among Children and Adolescents with Neurodevelopmental Disorders: A Practical Guide for Clinicians. World J. Clin. Pediatr..

[149] Mantle D., Smits M., Boss M., Miedema I., van Geijlswijk I. (2020). Efficacy and Safety of Supplemental Melatonin for Delayed Sleep–Wake Phase Disorder in Children: An Overview. Sleep Med. X.

[150] Li D., Smith D., Hardeland R., Yang M., Xu H., Zhang L., Yin H., Zhu Q. (2013). Melatonin Receptor Genes in Vertebrates. Int. J. Mol. Sci..

[151] Ahmad S.B., Ali A., Bilal M., Rashid S.M., Wani A.B., Bhat R.R., Rehman M.U. (2023). Melatonin and Health: Insights of Melatonin Action, Biological Functions, and Associated Disorders. Cell. Mol. Neurobiol..

[152] Sehirli A.O., Sayıner S., Chukwunyere U., Serakinci N. (2021). Role of Melatonin in Angiotensin and Aging. Molecules.

[153] Martín Giménez V.M., de las Heras N., Lahera V., Tresguerres J.A.F., Reiter R.J., Manucha W. (2022). Melatonin as an Anti-Aging Therapy for Age-Related Cardiovascular and Neurodegenerative Diseases. Front. Aging Neurosci..

[154] Erdem Y., Altunay İ., Özkur E., Şekerlisoy G., Karabay E., Özdemir F., Çerman A. (2021). The Association between Melatonin Levels and Sleep Quality in Patients with Pruritus: A Potential Biomarker on a Candidate Future Treatment. Indian J. Dermatol..

[155] Sabot D., Lovegrove R., Stapleton P. (2023). The Association between Sleep Quality and Telomere Length: A Systematic Literature Review. Brain Behav. Immun.-Health.

[156] Hasannia E., Derakhshanpour F., Vakili M.A. (2021). Effects of Melatonin on Salivary Levels of Cortisol and Sleep Quality of Hemodialysis Patients: A Randomized Clinical Trial. Iran. J. Psychiatry.

[157] Xie Y., Lou D., Zhang D. (2021). Melatonin Alleviates Age-Associated Endothelial Injury of Atherosclerosis via Regulating Telomere Function. J. Inflamm. Res..

[158] Liu R., Fu A., Hoffman A.E., Zheng T., Zhu Y. (2013). Melatonin Enhances DNA Repair Capacity Possibly by Affecting Genes Involved in DNA Damage Responsive Pathways. BMC Cell Biol..

[159] Yaşar P., Ayaz G., User S.D., Güpür G., Muyan M. (2017). Molecular Mechanism of Estrogen–Estrogen Receptor Signaling. Reprod. Med. Biol..

[160] Mal R., Magner A., David J., Datta J., Vallabhaneni M., Kassem M., Manouchehri J., Willingham N., Stover D., Vandeusen J. (2020). Estrogen Receptor Beta (ERβ): A Ligand Activated Tumor Suppressor. Front. Oncol..

[161] Taheri M., Ghafouri-Fard S., Najafi S., Kallenbach J., Keramatfar E., Atri Roozbahani G., Heidari Horestani M., Hussen B.M., Baniahmad A. (2022). Hormonal Regulation of Telomerase Activity and HTERT Expression in Steroid-Regulated Tissues and Cancer. Cancer Cell Int..

[162] Motlani V., Motlani G., Pamnani S., Sahu A., Acharya N. (2023). Changed Endocrinology in Postmenopausal Women: A Comprehensive View. Cureus.

[163] Tire B., Ozturk S. (2023). Potential Effects of Assisted Reproductive Technology on Telomere Length and Telomerase Activity in Human Oocytes and Early Embryos. J. Ovarian Res..

[164] Lin J., Kroenke C.H., Epel E., Kenna H.A., Wolkowitz O.M., Blackburn E., Rasgon N.L. (2011). Greater Endogenous Estrogen Exposure Is Associated with Longer Telomeres in Postmenopausal Women at Risk for Cognitive Decline. Brain Res..

[165] Park J., Hu R., Qian Y., Xiong S., El-Sabbagh A.S., Ibrahim M., Wang J., Xu Z., Chen Z., Song Q. (2024). Estrogen Counteracts Age-Related Decline in Beige Adipogenesis through the NAMPT-Regulated ER Stress Response. Nat. Aging.

[166] Gu D., Li J., Little J., Li H., Zhang X. (2020). Associations between Serum Sex Hormone Concentrations and Telomere Length among U.S. Adults, 1999–2002. J. Nutr. Health Aging.

[167] Marais G.A.B., Gaillard J.-M., Vieira C., Plotton I., Sanlaville D., Gueyffier F., Lemaitre J.-F. (2018). Sex Gap in Aging and Longevity: Can Sex Chromosomes Play a Role?. Biol. Sex Differ..

[168] Fait T. (2019). Menopause Hormone Therapy: Latest Developments and Clinical Practice. Drugs Context.

[169] Kuhl H. (2005). Pharmacology of Estrogens and Progestogens: Influence of Different Routes of Administration. Climacteric.

[170] Lee D.-C., Im J.-A., Kim J.-H., Lee H.-R., Shim J.-Y. (2005). Effect of Long-Term Hormone Therapy on Telomere Length in Postmenopausal Women. Yonsei Med. J..

[171] Bayne S., Li H., Jones M.E.E., Pinto A.R., van Sinderen M., Drummond A., Simpson E.R., Liu J.-P. (2011). Estrogen Deficiency Reversibly Induces Telomere Shortening in Mouse Granulosa Cells and Ovarian Aging in Vivo. Protein Cell.

[172] Kresovich J.K., Parks C.G., Sandler D.P., Taylor J.A. (2018). Reproductive History and Blood Cell Telomere Length. Aging.

[173] Entringer S., Epel E.S., Lin J., Blackburn E.H., Buss C., Simhan H.N., Wadhwa P.D. (2015). Maternal Estriol Concentrations in Early Gestation Predict Infant Telomere Length. J. Clin. Endocrinol. Metab..

[174] Bayne S., Jones M.E., Li H., Pinto A.R., Simpson E.R., Liu J.-P. (2008). Estrogen Deficiency Leads to Telomerase Inhibition, Telomere Shortening and Reduced Cell Proliferation in the Adrenal Gland of Mice. Cell Res..

[175] Shen J., Terry M.B., Liao Y., Gurvich I., Wang Q., Senie R.T., Santella R.M. (2012). Genetic Variation in Telomere Maintenance Genes, Telomere Length and Breast Cancer Risk. PLoS ONE.

[176] Duckworth A., Ruth K.S., Prague J.K., Russell A.-M., Almond H., Conway J., Beaumont R.N., Wood A.R., Martin S., Lunnon K. (2022). Study of the Associations between Short Telomeres, Sex Hormones and Pulmonary Fibrosis. medRxiv.

[177] Calado R.T., Yewdell W.T., Wilkerson K.L., Regal J.A., Kajigaya S., Young N.S. (2006). Sex Hormones Modulate the Length of Telomeres of Normal and Telomerase-Mutant Leukocytes through the Estrogen Receptor Pathway. Blood.

[178] WHO Endometriosis. https://www.who.int/news-room/fact-sheets/detail/endometriosis.

[179] Ilhan M., Battal A., Kaptaner B., Dogan A., Donmez F., Yilmaz M.A., Eroglu H. (2023). Exploring the Ameliorative Effects of *Hypericum scabrum* L. on a Surgically-Induced Endometriosis Rat Model and Its Phytochemical Profiling by LC-MS/MS. Farmacia.

[180] Ashfaq S., Can A. (2024). Danazol.

[181] Townsley D.M., Dumitriu B., Liu D., Biancotto A., Weinstein B., Chen C., Hardy N., Mihalek A.D., Lingala S., Kim Y.J. (2016). Danazol Treatment for Telomere Diseases. N. Engl. J. Med..

[182] Chambers D.C., Lutzky V.P., Apte S.H., Godbolt D., Feenstra J., Mackintosh J. (2020). Successful Treatment of Telomeropathy-related Interstitial Lung Disease with Immunosuppression and Danazol. Respirol. Case Rep..

[183] Córdova-Oriz I., Kohls G., Iglesias C., Polonio A.M., Chico-Sordo L., Toribio M., Meseguer M., Varela E., Pellicer A., García-Velasco J.A. (2023). A Randomized Controlled Intervention Trial with Danazol to Improve Telomeric and Fertility Parameters in Women with Diminished Ovarian Reserve: A Pilot Study. Women’s Health Rep..

[184] Assis V., de Sousa Neto I.V., Ribeiro F.M., de Cassia Marqueti R., Franco O.L., da Silva Aguiar S., Petriz B. (2022). The Emerging Role of the Aging Process and Exercise Training on the Crosstalk between Gut Microbiota and Telomere Length. Int. J. Environ. Res. Public Health.

[185] Giorgi C., Marchi S., Simoes I.C.M., Ren Z., Morciano G., Perrone M., Patalas-Krawczyk P., Borchard S., Jędrak P., Pierzynowska K. (2018). Mitochondria and Reactive Oxygen Species in Aging and Age-Related Diseases. Int. Rev. Cell Mol. Biol..

[186] Maldonado E., Morales-Pison S., Urbina F., Solari A. (2023). Aging Hallmarks and the Role of Oxidative Stress. Antioxidants.

[187] Voicu S.N., Scărlătescu A.I., Apetroaei M.-M., Nedea M.I., Blejan I.E., Udeanu D.I., Velescu B.Ș., Ghica M., Nedea O.A., Cobelschi C.P. (2024). Evaluation of Neuro-Hormonal Dynamics after the Administration of Probiotic Microbial Strains in a Murine Model of Hyperthyroidism. Nutrients.

[188] Patangia D.V., Anthony Ryan C., Dempsey E., Paul Ross R., Stanton C. (2022). Impact of Antibiotics on the Human Microbiome and Consequences for Host Health. Microbiologyopen.

[189] Chitulea P., Gherai R., Cheta C., Marin Negru T. (2022). The Role of Intravaginal Prebiotics in Controlling the Evolution of Uncomplicated Bacterial and Fungal Vaginal Infections. Farmacia.

[190] Willing B.P., Russell S.L., Finlay B.B. (2011). Shifting the Balance: Antibiotic Effects on Host–Microbiota Mutualism. Nat. Rev. Microbiol..

[191] Velescu B.Ș., Ilie M.I., Amzăr A.I., Lupașcu R.E., Marandiuc I.M., Apetroaei M.-M., Arsene A.L., Blejan E.I., Nedea O.A., Fistos T. (2023). Development and Experimental Evaluation of Some Silver Nanoparticles with Antimicrobial Potential. Processes.

[192] Strasser B., Wolters M., Weyh C., Krüger K., Ticinesi A. (2021). The Effects of Lifestyle and Diet on Gut Microbiota Composition, Inflammation and Muscle Performance in Our Aging Society. Nutrients.

[193] Vyas C.M., Ogata S., Reynolds C.F., Mischoulon D., Chang G., Cook N.R., Manson J.E., Crous-Bou M., De Vivo I., Okereke O.I. (2021). Telomere Length and Its Relationships with Lifestyle and Behavioural Factors: Variations by Sex and Race/Ethnicity. Age Ageing.

[194] Hoffmann A., Kleniewska P., Pawliczak R. (2021). Antioxidative Activity of Probiotics. Arch. Med. Sci..

[195] Wang Y., Wu Y., Wang Y., Xu H., Mei X., Yu D., Wang Y., Li W. (2017). Antioxidant Properties of Probiotic Bacteria. Nutrients.

[196] Plaza-Diaz J., Ruiz-Ojeda F.J., Gil-Campos M., Gil A. (2019). Mechanisms of Action of Probiotics. Adv. Nutr..

[197] Amzăr A.I., Udeanu D.I., Pițuru M.T., Hîrjău M., Popa D.E., Velescu B.Ș., Letiția A. (2022). Signalling Through the Microbiota-Gut-Brain Triade. Farmacia.

[198] Zhang N., Zhang Y., Li M., Wang W., Liu Z., Xi C., Huang X., Liu J., Huang J., Tian D. (2020). Efficacy of Probiotics on Stress in Healthy Volunteers: A Systematic Review and Meta-analysis Based on Randomized Controlled Trials. Brain Behav..

[199] Messaoudi M., Violle N., Bisson J.-F., Desor D., Javelot H., Rougeot C. (2011). Beneficial Psychological Effects of a Probiotic Formulation (*Lactobacillus helveticus* R0052 and *Bifidobacterium longum* R0175) in Healthy Human Volunteers. Gut Microbes.

[200] Makarenko M.S., Chistyakov V.A., Usatov A.V., Mazanko M.S., Prazdnova E.V., Bren A.B., Gorlov I.F., Komarova Z.B., Chikindas M.L. (2019). The Impact of Bacillus Subtilis KATMIRA1933 Supplementation on Telomere Length and Mitochondrial DNA Damage of Laying Hens. Probiotics Antimicrob. Proteins.

[201] Gupta A., Sharma S., Reichenbach P., Marjavaara L., Nilsson A.K., Lingner J., Chabes A., Rothstein R., Chang M. (2013). Telomere Length Homeostasis Responds to Changes in Intracellular DNTP Pools. Genetics.

[202] Lew L.C., Hor Y.Y., Jaafar M.H., Lau A.S.Y., Ong J.S., Chuah L.O., Yap K.P., Azzam G., Azlan A., Liong M.T. (2019). Lactobacilli Modulated AMPK Activity and Prevented Telomere Shortening in Ageing Rats. Benef. Microbes.

[203] Shan S., Zheng T., Zhang C., Song X., Chen J., Shi Y., You J., Cheng G., Xiong J. (2020). Yogurt and Streptococcus Thermophilus Metabolites Ameliorated Telomere Attrition in D-galactose-induced Ageing Mice and t -BHP-challenged HepG2 Cells. Int. J. Food Sci. Technol..

[204] Matsumoto M., Kurihara S., Kibe R., Ashida H., Benno Y. (2011). Longevity in Mice Is Promoted by Probiotic-Induced Suppression of Colonic Senescence Dependent on Upregulation of Gut Bacterial Polyamine Production. PLoS ONE.

[205] Hunsche C., Cruces J., De la Fuente M. (2019). Improvement of Redox State and Functions of Immune Cells as Well as of Behavioral Response in Aged Mice After Two-Week Supplementation of Fermented Milk with Probiotics. Curr. Microbiol..

